# Obesity and food marketing: a narrative review of consumer influence, regulatory gaps, and ethical implications

**DOI:** 10.3389/fnut.2025.1645166

**Published:** 2025-08-29

**Authors:** Anam Farzand, Mohd Adzim Khalili Rohin, Sana Javaid Awan, Ahram Hussain, Muhammad Faizan, Abdul Momin Rizwan Ahmad

**Affiliations:** ^1^Faculty of Health Sciences, School of Nutrition and Dietetics, Universiti Sultan Zainal Abidin (UNISZA), Terengganu, Malaysia; ^2^Institute of Molecular Biology and Biotechnology (IMBB), The University of Lahore, Lahore, Pakistan; ^3^Department of Medical Laboratory Technology, Superior University, Lahore, Pakistan; ^4^Institute of Microbiology, University of Veterinary and Animal Sciences, Lahore, Pakistan; ^5^Department of Health Sciences, University of York, York, United Kingdom; ^6^Department of Human Nutrition and Dietetics, NUST School of Health Sciences, National University of Sciences & Technology (NUST), Sector H-12, Islamabad, Pakistan

**Keywords:** obesity, food marketing, food industry, agriculture, consumer choices, regulatory frameworks, social cognitive theory, REFCAM

## Abstract

**Background:**

Obesity is a multifactorial global health crisis exacerbated by modern food marketing strategies that encourage the consumption of energy-dense, nutrient-poor (EDNP) foods. Children and socio-economically disadvantages groups are particularly vulnerable to the cognitive and emotional cues embedded in food advertising.

**Objectives:**

To investigate the ethical aspects of marketing to vulnerable groups, evaluate the shortcomings of the current regulatory frameworks, critically analyze the impact of food marketing on consumer behavior and dietary patterns, and offer policy-relevant insights for public health interventions. Methods: Multidisciplinary sources from the fields of public health, behavioral psychology, marketing science, and nutrition policy were synthesized through an extensive narrative review. Through methodical searches of databases such as PubMed, Scopus, Web of Science, and Google Scholar, peer-reviewed and gray literature were found. Among the theoretical models used are the REFCAM model, the Theory of Planned Behavior, and the Social Cognitive Theory.

**Results:**

Food marketing uses digital microtargeting, sensory cues, and psychological priming to influence consumption. Overconsumption has become commonplace due to strategies including portion control, manipulating brand loyalty, and health halo benefits. Despite global variation in regulatory responses, corporate lobbying, disjointed governance, and inadequate digital oversight often limit their effectiveness. Marketing aimed at minorities and children raises ethical concerns, as there is proof of exploitation through deceptive and culturally specific advertising. Comparative case studies highlight regulatory achievements (like Chile and France) as well as failures (like UK policy delays).

**Conclusion:**

Food marketing is a major contributor to the development of obesogenic environments, despite being poorly controlled. A change from reactive to proactive, system-level governance is necessary to combat obesity. Strong digital control, more stringent nutrient profiling, and a moral shift in food marketing strategies are all part of this. Promoting healthy choices and safeguarding vulnerable people require cross-sectoral collaboration.

## 1 Introduction to obesity and food marketing

According to the World Health Organization (WHO), obesity is a complicated, multifaceted chronic condition that poses a risk to one’s health due to an excessive or aberrant buildup of body fat. Clinical diagnosis of obesity is typically based on Body Mass Index (BMI ≥ 30 kg/m^2^). However, additional anthropometric measures such as waist circumference and waist-to-hip ratio are increasingly used to evaluate central adiposity and associated metabolic risk (1). The obesogenic environment, which encourages excessive calorie intake and sedentary lifestyles, is receiving more attention even though obesity is caused by a combination of genetic, metabolic, behavioral, and environmental variables. Food marketing is a significant factor in this environment, since it disproportionately promotes energy-dense, nutrient-poor (EDNP) goods, especially to kids and teenagers. Ultimately, this commercial influence impacts public health by influencing consumption patterns and preferences. Therefore, this book explores how deliberate food marketing strategies reinforce unhealthy eating habits and behavior’s, thereby exacerbating the global obesity issue.

**Figure 1:**
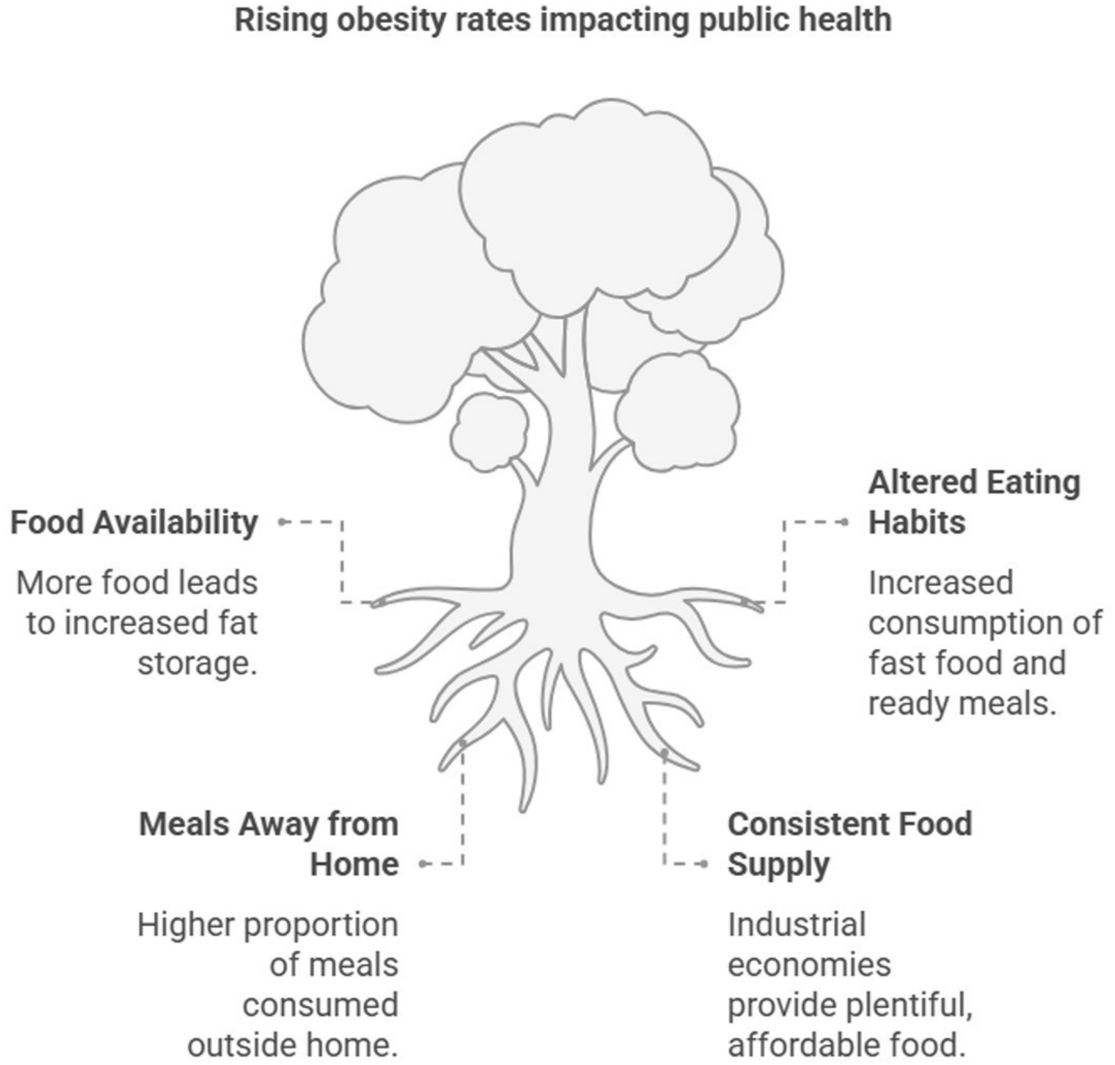
Rising obesity rates impacting public health.

Obesity has been recognized as a medical concern since the advent of clinical diagnosis and epidemiological observation. According to the World Health Organization, it has reached an epidemic progression in recent decades (1, 2). This epidemic has a profound effect on public health because obesity, particularly intra-abdominal fat mass, is linked to a dysregulation of lipoprotein-lipid metabolism and several pathologies such as coronary heart disease, type 2 diabetes, liver disease, cancers, sleep disordered breathing disorders (both obesity hypoventilation syndrome and obstructive sleep apnea), gastroesophageal reflux disease, gallstones, pancreatitis and osteoarthritis and respiratory system disorders (3–6). Today, we have more food available and, hence, more energy to store; thus, the capacity of people to store fat has increased accordingly. The existence of metabolically healthy obese individuals, alongside metabolically unhealthy individuals of normal weight, challenges the conventional classification of obesity as a disease, suggesting that not all obese individuals experience adverse health outcomes (7, 8). The fast-food sector is confronted with a mature market with minimal scope for expansion. Eating habits have altered significantly over the last few decades. Fast food restaurants, frozen foods, and ready meals in supermarkets now provide consumers with alternative options that were not as ubiquitous in the past. The proportion of meals consumed away from home has been consistently growing, contributing between 40 and 50% of total meals consumed. The food supply in industrial economies tends to be consistent, affordable, and plentiful. Due to these consumer food preferences, the consumer food channel structure has drastically changed over the past three decades concerning providing food to consumers, where and when food is eaten, and by whom (9). In the modern era, food marketing affects what and how much people eat. More and more research indicate that marketing tactics frequently encourage habitual overconsumption by promoting greater portions, especially for snacks, fast food, and beverages with added sugar (10). This condition, which is known as “portion distortion,” has made consuming too many calories acceptable, particularly for kids and teenagers (Figure 1).

Marketing Strategy is something that assists companies in accomplishing Marketing goals. Marketing goals help achieve corporate goals, and corporate goals strive to attain a competitive edge over competing organizations. Within this article, we attempt to present the meaning and definition of marketing strategy; the history of Marketing Strategy; Development of Marketing Strategy; Framework for Marketing Strategy (11, 12). One of the novel theoretical models for explaining the covert mechanism of ill food promotion is the REFCAM (Reactivity to embedded food cues in advertising model). The REFCAM combines, alongside other theoretical approaches, the commercialized media content processing model and the differential susceptibility to media influence model, and is based on three key assumptions (12–14). According to the REFCAM, it is particularly promising to study how promotion of healthy food can assist in enhancing consumption of foods or food groups delivering needed nutrients, preventing chronic disease, and encouraging overall health, e.g., fruit or vegetables (12).

A multidisciplinary approach is taken in this narrative review to comprehend the role that food marketing plays in the obesity pandemic (15). The work is organized thematically and conceptually, with each central section focusing on a different sphere of influence, such as advertising methods, consumer behavior, regulatory contexts, ethical considerations, and upcoming technology. Nonetheless, we admit that certain conceptual similarities might have made some parts seem less distinct, particularly in the introduction. The Black Box model, REFCAM, the Country-of-Origin Effect, the Social Cognitive Theory, the Theory of Planned Behavior, the Black Box model, and the Consumer Decision Process Model were all used to enhance the theoretical foundation. Their selection aimed to reflect complementary viewpoints on the cognitive, behavioral, sociological, and commercial aspects of food marketing. By addressing distinct aspects of consumer behavior and marketing influence, each theory contributes to a more comprehensive understanding of the formation of obesogenic environments. Because the relationship between food marketing and obesity is complex and involves psychological, social, and structural factors, it was decided to include different frameworks. Instead, we employed these frameworks to support the story around several axes of influence that converge in modern food marketing strategies (16).

### 1.1 Aims and objective

This narrative review aims to critically analyze the complex connection between food marketing tactics and the worldwide obesity crisis.

In particular, it aims to:

- Examine the effects of marketing strategies on consumer behavior across various groups, including branding, portion control, digital targeting, and packaging.
- Examine how regulatory frameworks can mitigate obesogenic conditions, paying particular attention to differences between high- and low-income (LMIC) and low-income nations.
- Examine the moral ramifications of food marketing, especially concerning vulnerable groups like children.
- Analyze how new technology and digital platforms influence dietary habits and food advertising practices.

This review attempts to provide policy-relevant insights that can guide successful strategies to reduce obesity at the population level by combining theoretical models, international case studies, and regulatory assessments.

### 1.2 Methodology of literature review

The intricate connection between food marketing strategies and the worldwide increase in obesity is examined in this article’s thorough narrative literature analysis, emphasizing vulnerable groups like children. The narrative review methodology was chosen because it analyzes wide-ranging, multidisciplinary subjects across media studies, public Health, consumer behavior, and nutrition policy. The experts used a methodical search approach to locate pertinent material to ensure a comprehensive topic investigation. We searched databases such as PubMed, Scopus, Web of Science, and Google Scholar by combining keywords like “obesity,” “food marketing,” “consumer behavior,” “children,” “fast food,” “advertising,” and “policy.” Both peer-reviewed research publications and gray literature—such as policy reports from agencies like the CDC and WHO—as well as industry data, were included in the literature selection. According to the inclusion criteria, studies examining how marketing affects eating habits and obesity were given preference, particularly if they offered empirical data, theoretical viewpoints, or regulatory consequences.

After a thorough full-text review, the gathered studies were filtered by title and abstract. To create a conceptual synthesis across several dimensions, such as the mechanisms of food advertising, consumer behavior models (such as the Social Cognitive Theory, Theory of Planned Behavior, and REFCAM), branding and packaging strategies, regulatory approaches, ethical considerations, and the role of emerging technologies in food marketing, the articles were then thematically categorized (17, 18). By arranging the information, the writers could present a comprehensive yet coherent picture of how marketing tactics lead to developing surroundings conducive to obesity. The manuscript’s main objective is to provide a thorough and multidisciplinary analysis that explains how food marketing affects consumer decisions and leads to unhealthy eating habits. It guides how these practices can be addressed through well-informed public health and policy interventions. Despite being a narrative review, the writers followed a methodical approach while choosing the literature, guaranteeing methodological soundness and consistency. The study integrates academic theories with policy evaluations and case studies, drawing on conceptual frameworks and real-world experiences. A more thorough understanding of how marketing strategies, particularly those aimed at youngsters, influence dietary decisions and fuel the obesity epidemic is made possible by this hybrid approach. The ultimate goal is to contribute to academic discussions and aid in creating successful programs and regulations meant to reduce obesity by tackling the systemic causes of inadequate nutrition.

This review aims to compile and assess multidisciplinary research on the connection between food marketing and consumer behavior, especially concerning obesity. This paper examines the following topics using theoretical frameworks such as the Social Cognitive Theory, Theory of Planned Behavior, and Dual Process Models: How specific marketing strategies, like incidental signals, persuasive design, and health halos, impact consumer decision-making; How marketing influence is moderated by sociodemographic factors (e.g., sex, age, BMI, and socio-economic status); foundations for regulations and policies created to lessen the adverse health effects of aggressive food marketing, emphasizing global models like the General Food Law of the European Union. This review offers a thorough and policy-relevant knowledge of how food marketing contributes to the obesogenic environment by combining behavioral science, marketing, and public health findings.

## 2 Theoretical frameworks in consumer behavior

**Figure 2:**
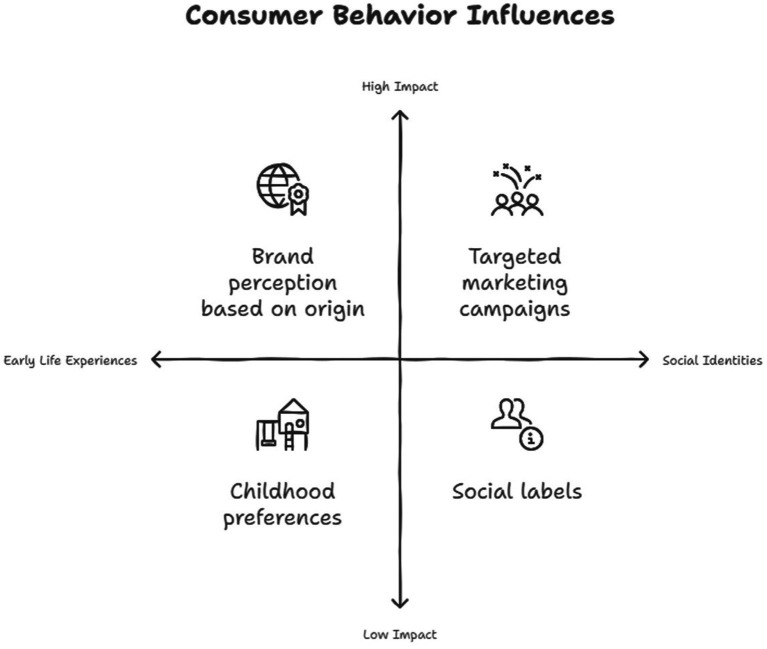
Consumer behavior influence.

Marketing researchers have appreciated the significance of early life experiences in shaping consumer behavior patterns at later ages. Still, they have had insufficient theoretical and methodological foundations to address these consumer behavior issues in life course settings. Research identifying age differences does not indicate consumer behavior changes or experience impacts on current consumer behavior patterns, since individual consumers or groups are not analyzed compared to past experiences or life stages within historical and cultural contexts (19). Country-of-Origin (CoO) is a marketing technique that incorporates national origin cues into branding and positioning so consumers can use perceived national stereotypes to infer product traits like quality, authenticity, or status. For instance, Japanese products tend to be thought of as high-quality, while Swiss products tend to be seen as precise. CoO remains significant in product assessments because it determines pre-purchase judgments (20). Consumers tend to be targeted, profiled, addressed, or overlooked in the market based on elements of their social identities (e.g., athlete, environmentalist, student, age, gender, race). These social labels are constructed based on features that distinguish individuals as different or distinct from other consumers and can provide marketers with an easy means of structuring the marketplace or speaking to a particular segment of consumers, as when department stores address consumers by age or gender (21). With many social and environmental factors contributing to its rising prevalence, obesity has become more widespread (Figure 2).

**Figure 3:**
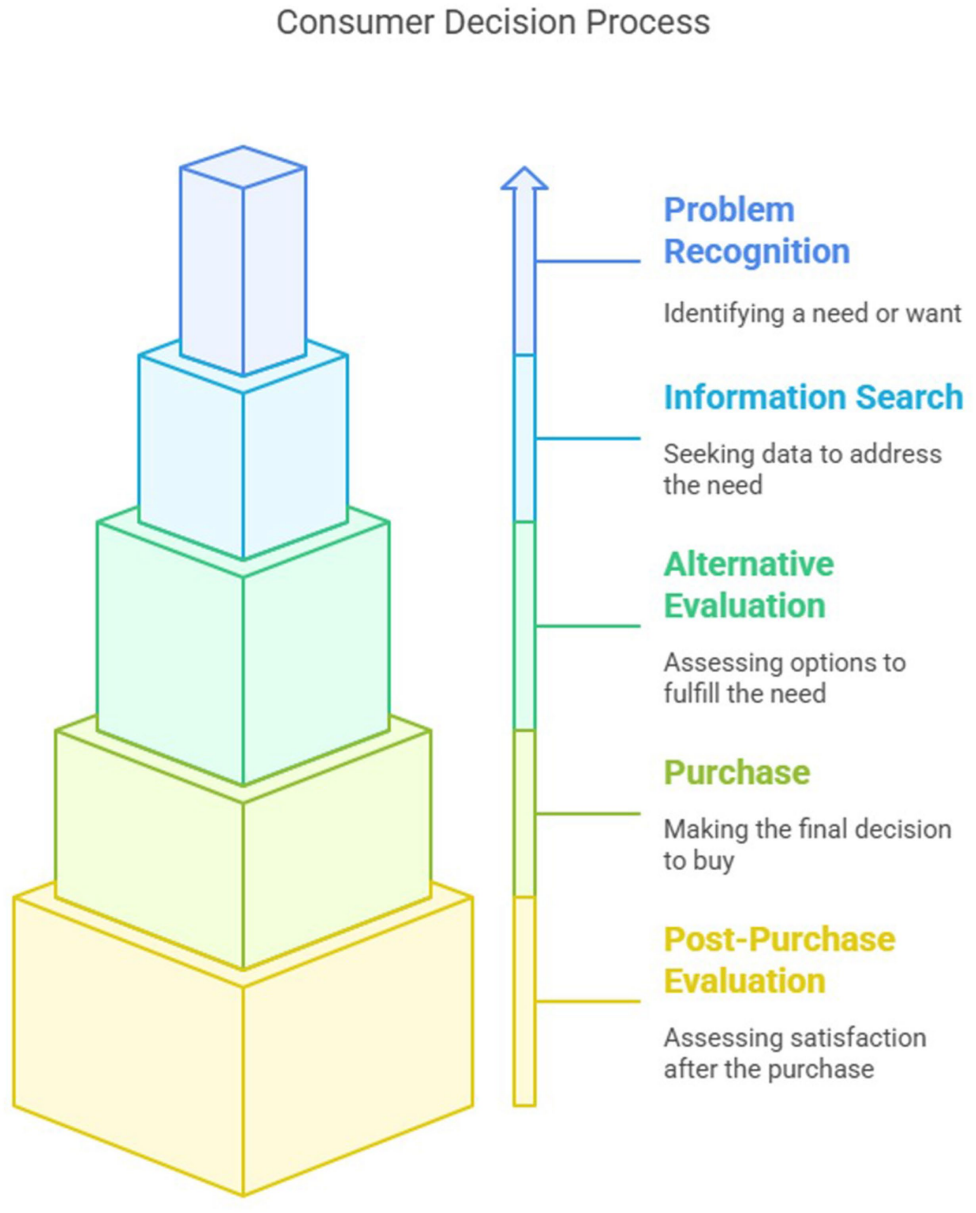
Consumer decision process.

The basic model used for consumer choice is the consumer decision process. It identifies five stages representing consumer’s complete activities when purchasing goods and services. These include (1) problem recognition, (2) information search, (3) alternative evaluation, (4) purchase, and (5) post-purchase evaluation (22, 23). Today, the “black box” model of consumer behavior is the most widely recognized and used by marketers compared to alternatives. Marketers acknowledge that consumer decision-making is a complex process involving multiple and interrelated influences (24). The “black box” model captures this complexity by proposing that a “box” separates the consumer’s environment, which creates the need, from the consumer’s response to that need, the purchase (24). The consumer’s characteristics and decision process are captured within the box. Marketers have long been interested in which variables inside the black box can be influenced and to what extent. For example, products and brands are often labeled to imply quality, provide a guarantee, or a measure of safety and reassurance, regardless of whether those attributes are provided or not. Business and non-business organizations must understand the black box as an undertaking to influence it (24, 25) (Figure 3).

### 2.1 Social cognitive theory

**Figure 4:**
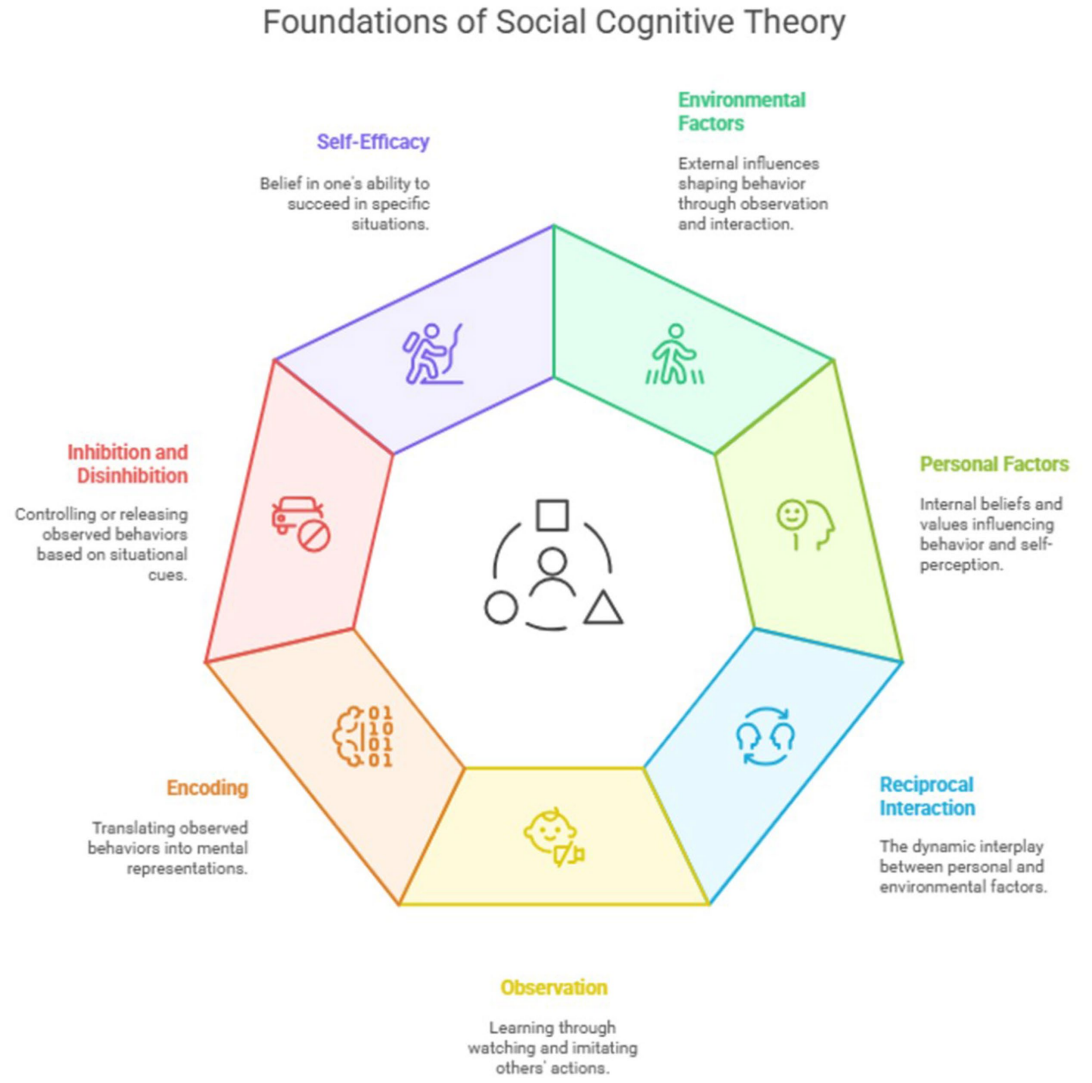
Foundation of social cognitive theory.

Psychologist Albert Bandura developed Social Cognitive Theory. The theory defines how individuals acquire and maintain behaviors through environmental factors, personal factors, and the reciprocal interaction of both (17, 26). In this model, personal factors affect the individual, the value they put on themselves, and the environment. This occurs frequently in such a way that feedback flows from the individual and their environment. Bandura lists several personal factors that influence human behavior, but research has shown that these personal factors do not influence obese behavior; hence, it is not of interest in this study (17). Constructed as an extension of Social Learning Theory, Bandura described the SCT (Social Cognitive Theory) as a social learning process that can occur purely based on observation, without any associated learning or direct reinforcement. The SCT holds three fundamental activities in the learning process: (1) Observation: An individual’s behavior is influenced by the observed behavior of another person. (2) Encoding: The observed behavior is then translated into a mental representation; specifically, a joint representation of the situation. (3) Inhibition and disinhibition: The third step discuss the inhibition or disinhibition of observed behavior (27). Once the mental representation of the joint situation has been established, an agreement should be made, mediated by situational cues that provide evidence of whether or not the observed behavior is approved. This usually involves observing the consequences of the observed behavior. Additionally, self-efficacy is one of the principal concepts in SCT and one of the foundations of SC theory (17, 18) (Figure 4).

### 2.2 Theory of planned behavior

Theory of planned behavior (TPB) has been employed effectively to explain and predict behavior in a wide range of behavioral contexts, from physical activity to drug consumption, from recycling to mode of travel choice, from safer sex to consumerism, and from adoption of technology to privacy protection (28, 29). TPB begins with an outright definition of the target behavior in terms of the target, behavior to be involved in, where and when the behavior is engaged, and the period concerned. These dimensions can each be defined on some level of specificity or generality. For TPB, attitudes toward behavior, subjective norm toward behavior, and perceived control of behavior are used to determine intentions to engage in a behavior (29). The Theory of Planned Behavior adds perceived behavioral control to the Theory of Reasoned Action. The Theory of Planned Behavior asserts that a behavior is determined by an individual’s intentions and perceived behavioral control. Perceived behavioral control or self-efficacy refers to the degree to which an individual feels they are in control of doing that behavior (30). There are three basic pre-postulates of TPB. One is behavioral intention. Behavioral intention is the extent to which a person is willing to perform, or carry out, a specific behavior. It is more than mere willingness, as it is determined by desire and motivation, and intent implies that the individual plans and expects to perform the behavior. In essence, intention measures how hard people are willing to try and the effort they are willing to exert to perform said behavior (31, 32).

### 2.3 Comparative analysis of behavioral frameworks: SCT, TPB and REFCAM

A multifaceted theoretical approach is necessary to comprehend how food marketing affects consumer behavior, particularly in obesogenic situations. This review is based on three major models: the Reactivity to Embedded Food Cues in Advertising Model (REFCAM), the Theory of Planned Behavior (TPB), and the Social Cognitive Theory (SCT). Each offers unique, yet complementary, perspectives on how people react to food marketing cues (Table 1).

**Table 1:** Comparative analysis of frameworks.

Framework	Core construct	Relevance of food marketing	Unique contribution
SCT	Observational learning, Self-efficacy, Reciprocal determinism	Explains how modeling and reinforcement from regular exposure to food commercials affect eating habits.	Highlights how social modeling plays a part in habit formation, particularly in children and adolescents.
TPB	Attitude, Subjective norms, Perceived behavioral control, Intention	Shows how marketing influences purchase intent by changing attitudes and perceived norms around food intake.	Provides the ability to foresee how intentions will mediate between beliefs and actions.
REFCAM	Attentional bias, Cognitive elaboration, Emotional reactivity	Explains how food cues incorporated into advertisements cause instinctive, emotional reactions that circumvent logical judgment.	Captures unconscious mechanisms that are frequently abused in contemporary digital food marketing.

**Figure 5:**
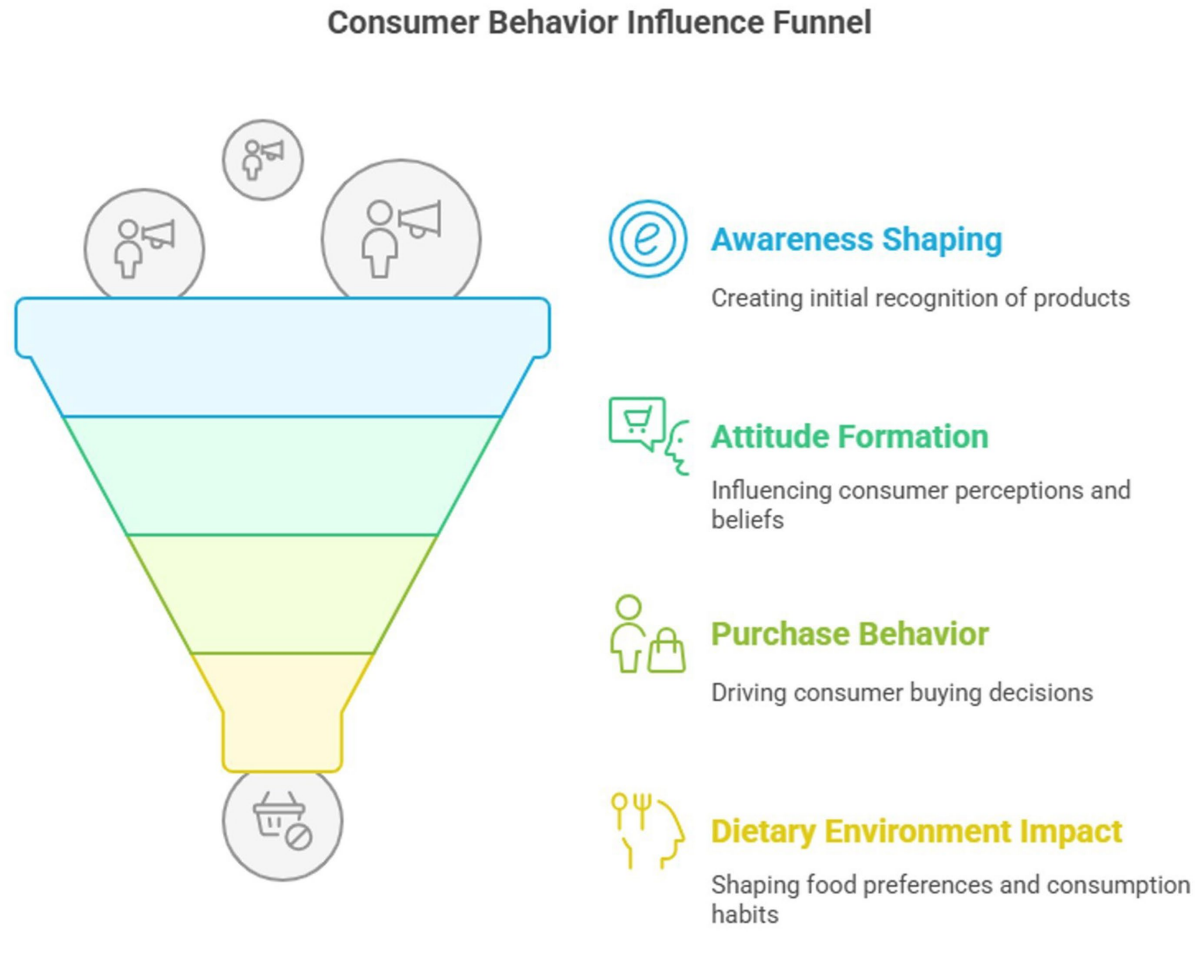
Theory of planned behavior framework.

REFCAM emphasizes the automaticity of marketing impact, which is especially important in digital and advergaming environments because consumers, especially children, are less cognitively defensive. SCT and TPB place more emphasis on intentional and socially aware behavior modification. Combined, these models offer a more thorough and nuanced understanding of how food marketing can influence consumer decisions covertly and explicitly. A more thorough understanding of the psychological workings of marketing is made possible by this integrated viewpoint, which also facilitates the development of multi-tiered interventions, from systemic policy changes to behavioral assistance at the individual level (33) (Figure 5).

## 3 Impact of food advertising on consumer preferences

Portion control is an essential but frequently overlooked aspect of food marketing that greatly aids in creating obesogenic environments. The practice of “supersizing,” which gained popularity in the 1990s, entails raising portions at little extra expense to the customer, especially for fast food, snack foods, and beverages with added sugar. This encourages overconsumption while boosting unit sales volume. Packaging design is also essential; “portion distortion” is the term used to describe how visual signals and container proportions frequently skew perceptions of proper serving quantities. Research shows that regardless of hunger or satiety signs, people consistently eat more when given larger quantities. This effect is particularly noticeable in children and adolescents because of their inability to control their consumption (34). These practices have weakened public health initiatives advocating moderation, which have also normalized excessive portion sizes and changed societal norms surrounding acceptable intake. It has been demonstrated through controlled trials that exposure to bigger portion sizes can result in long-term increases in daily calorie intake, which can directly contribute to weight gain and positive energy balance over time. Effective treatments aiming at recalibrating portion standards and restoring consumer autonomy require understanding portion manipulation as a purposeful marketing strategy (35). Advertising, public relations, and sales promotion are mass-communication techniques at marketers’ disposal. As its name indicates, mass communication employs the same message for all members of an audience. Mass communication techniques sacrifice the benefit of personal selling, the ability to customize a message for each prospect, and the benefit of covering large numbers of people at a lower cost. An Individual Advertiser’s primary goal is to reach potential customers and shape their awareness, attitudes, and purchase behavior. They spend a lot of money to keep people (markets) interested in their products. To be successful, they must learn what makes prospective customers act like they do (36).

**Figure 6:**
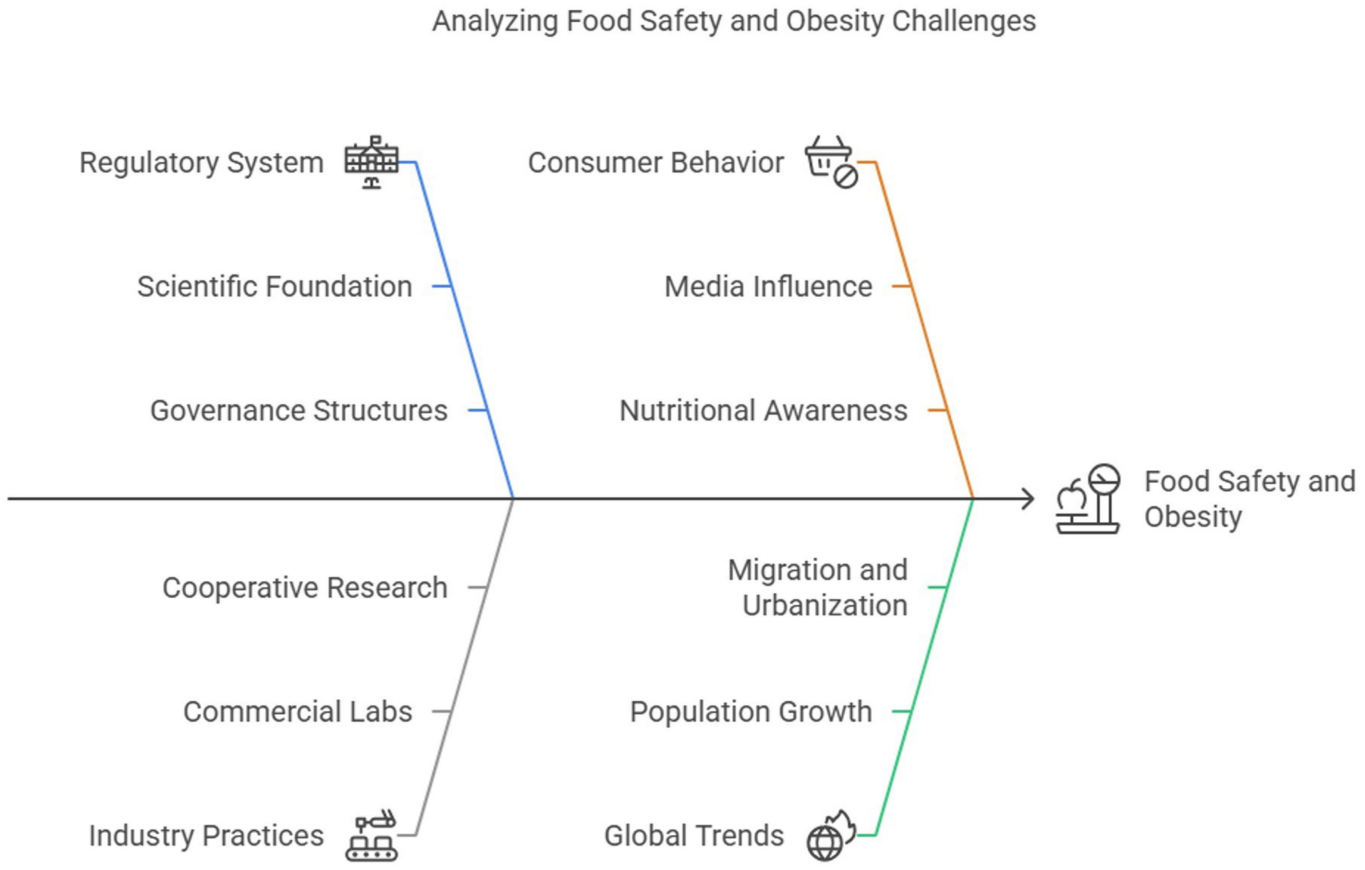
Consumer behavior influence funnel.

Numerous stimuli affect consumer behavior, which studies consumer buying habits. There are several steps a consumer undergoes before they purchase products found in the market. Cultural, social, personal, or psychological factors affect people’s buying decisions (37). Heavy advertising of nutrient-poor foods results in increased preference for these products and a change in the volume consumed of high-fat and high-energy foods. High exposure rates to advertising are also positively related to the probability of consuming foods whose images are aired in the advertisements (38). This dietary environment is the only one all our generation of youth has ever known; they are high users of soft drinks, snack foods, and fast food, and more at risk for being overweight than ever before (39, 40). The public image of this poisoned food environment is food advertising. Widespread marketing of unhealthy foods creates social norms around acceptable and desirable foods. Ads are conditioned stimuli that activate cravings for food and boost food intake, especially in children (41, 42) (Figure 6).

### 3.1 Effects of targeted marketing on vulnerable populations

Today’s advertising relies heavily on targeted marketing tactics, which choose customers who share traits or demands and target them with certain products. Although the United States’ history of ethnic targeting in food advertising dates back to the 1980s and early 1990s, more recent patterns show that ethnic identities are still being used through digital media in a persistent and increasingly complex way (43). According to 2021 research by the Rudd Centre for Food Policy and Health, ads for fast food, sugar-sweetened beverages, and snack goods continue to be disproportionately shown to Black and Hispanic kids in the US on digital and television media. These advertisements frequently use language, music, and imagery that are culturally appropriate and intended to appeal to particular ethnic groups to boost brand engagement and buy intention (44). Multinational food companies in Brazil and South Africa have also used influencer relationships, culturally relevant narratives, and geodemographic profiling to promote ultra-processed meals to low-income ethnic groups and metropolitan areas. Even though these marketing strategies are presented as inclusive or culturally sensitive, they frequently worsen health inequities by pushing nutrient-dense, high-energy meals on groups that are already at a higher risk of obesity and diet-related illnesses. The need to regulate marketing strategies that target vulnerable ethnic populations under the pretense of cultural significance is made more urgent by this changing environment (45, 46).

Food marketing strategies in low- and middle-income countries (LMICs) increasingly take advantage of urbanization, growing digital access, and lax regulatory monitoring. The widespread use of mobile devices has made it possible for aggressive digital advertising to target children and teenagers with highly processed and energy-dense meals, particularly through social media and gaming platforms. Child-directed food marketing has increased in nations like India, Nigeria, and Pakistan, frequently with no legal limits. Transnational fast-food chains use culturally relevant marketing in these situations (e.g., ethnic flavors, local language campaigns) to elicit strong feelings from customers while disguising nutritional issues.

Furthermore, where industry self-regulation does exist, it is typically unenforceable. Socioeconomically disadvantaged populations are more vulnerable as a result of these structural gaps, which further exacerbate nutritional disparities and hasten the transition from undernutrition to obesity (47, 48). Children’s behavioral responses often differ from those of adults following food advertising exposure. Children appear less resistant or skeptical in their response to advertising than adults because they generally believe that advertising claims are valid (49). Children who frequently view television food advertising are strongly influenced by their parents, which determines their purchasing behavior (13, 49, 50).

Recent research shows that food marketing deliberately impacts dietary behavior by focusing on psychological and emotional decision-making pathways, rather than just informing consumers. This is important given the rising incidence of obesity worldwide. Marketing tactics frequently use automatic cognitive processes, visual stimuli, emotional appeals, and priming cues to encourage impulsive buying, especially in susceptible demographics like children, teenagers, and the impoverished. These marketing strategies are rarely neutral; instead, they consistently raise brand loyalty, normalize overconsumption, and change perception thresholds—even when nutritional quality is subpar. Crucially, people’s reactions to food ads are strongly influenced by sociodemographic modifiers like age, sex, and socio-economic position. For example, individuals in lower income levels might rely on price signals and convenience aspects, but children are more receptive to colorful packaging and compelling cartoon characters. It is essential to acknowledge these varied susceptibilities to design successful treatments that target both commercial drivers and behavioral disparities in food choices (51).

### 3.2 Influence of packaging and branding

Researchers and practitioners must understand how important sensory qualities such as taste, texture, scent, and esthetic appeal are in influencing consumer attitudes and purchase intentions. To strengthen the appeal of a product, these organoleptic qualities work in tandem with non-sensory cues like branding or labeling. For example, the crispness of apples or the crunchiness of fresh vegetables are frequently linked to the perception of freshness and quality, which significantly impacts consumers repurchase behavior. Subtle variations influence consumer choice and brand loyalty in mouthfeel, aromatic characteristics, and flavor complexity in the wine and cheese industries (52, 53). Marketing strategies often utilize these sensory signals to augment hedonic attractiveness. Studies indicate that aromatic chemicals in coffee and baked goods can elicit emotional reactions and stimulate appetites, irrespective of hunger. Furthermore, food makers may create hyperpalatable items by manipulating combinations of fat, sugar, and salt or by modifying texture through additives to encourage overconsumption, thereby adding to obesogenic environments (54). Packaging serves a crucial dual purpose as well. Functionally, it preserves the integrity of the product and increases shelf life. It raises sensory expectations from a marketing standpoint by using visual design (glossy finish, color schemes), imagery (freshness cues), and even audio signals (sound of opening a fizzy beverage). Together, these design components affect consumers’ expectations of taste and quality before tasting the product (55). Therefore, in today’s food contexts, it is crucial to comprehend and maximize the interaction of sensory and extrinsic elements to influence consumer food choices.

According to data from past research, the number of cross-promotions on food packaging targeted at children in the United States increased significantly, increasing by 78% between 2006 and 2008; however, only 18% of those products fulfilled recognized nutritional requirements (55). Even while these results are still significant historically, more recent studies have shown that nutrient profiling requirements are frequently not followed in child-targeted marketing, which further supports the issue’s enduring nature (56, 57). In the modern consumer economy, well-known brands are essential because they are seen as trustworthy, recognizable, and high-quality. Because of their emotional connections or prior pleasant experiences, consumers are frequently prepared to pay more for branded products. Though this brand loyalty benefits producers and merchants, it also raises questions about how it might limit customer choice and hamper competition, mainly when it’s used to conceal subpar products or avoid making educated decisions (58). Customers may replace objective quality evaluation with brand reputation, according to a wealth of evidence, particularly in low-involvement or fast-moving consumer goods (FMCG) sectors. Although this could simplify decision-making, it exposes customers to deceptive marketing strategies prioritizing perceived value over real nutritional or health advantages. Crucially, these factors could worsen health disparities since those with few resources might choose highly marketed, low-nutrition products that receive the most advertising. This raises significant moral and legal issues: Is brand loyalty a tacit kind of behavioral control, or should it be seen as a means of customer empowerment? To what Degree should marketing rules step in to safeguard consumer autonomy from prejudices influenced by brands? (59).

## 4 Regulatory environment and policy implications

The regulatory system, with all the factors that constitute it scientists, consumers, industry, and Congress determines safety in the context of modern technology, scientific ability, and the level of tolerance of the lay public. Scientific foundation becomes extremely critical as more and more businesses shift away from maintaining in-house research facilities toward using commercial laboratories and universities, as well as engaging in cooperative research efforts to address food safety and product development research requirements (60). Food regulation primarily seeks to safeguard the consumer’s Health, enhance economic viability, harmonize well-being, and promote equitable trade in foods among and within countries. It is necessary to weigh the advantage of expanded food supplies through technology against related health and economic threats, a need that is ever more critical considering the increasing pattern of world population, opening of borders, migration, urbanization, and accompanying changing food habits (61). Governance structures form the foundation for planning, implementing, and assessing regulatory interventions. Australia has a multitiered system of governance with more than 560 local councils, eight state/territory parliaments, the federal parliament, and connections to the international system (62, 63).

Several nations have enacted specific laws to curb food marketing and product use to lower obesity rates. For example, Mexico’s 2014 tax on sugar-sweetened beverages (SSBs) reduced soda consumption by 12% in the first year, with low-income households experiencing the most significant impact (64). Similarly, Chile drastically changed food marketing tactics in 2016 when it enacted extensive food labeling regulations and marketing limitations, such as prohibiting children’s promotion of high-sugar goods (65). The difficulties in putting policy into practice are shown by the fact that, in 2021, England declared a ban on junk food advertising on TV and online platforms before 9 p.m. Still, industry lobbying and legislative resistance have delayed its implementation (66). These instances highlight the necessity of strong, enforced rules to successfully reduce obesogenic surroundings and show the intricate relationship between public health priorities, political will, and industry resistance.

**Figure 7:**
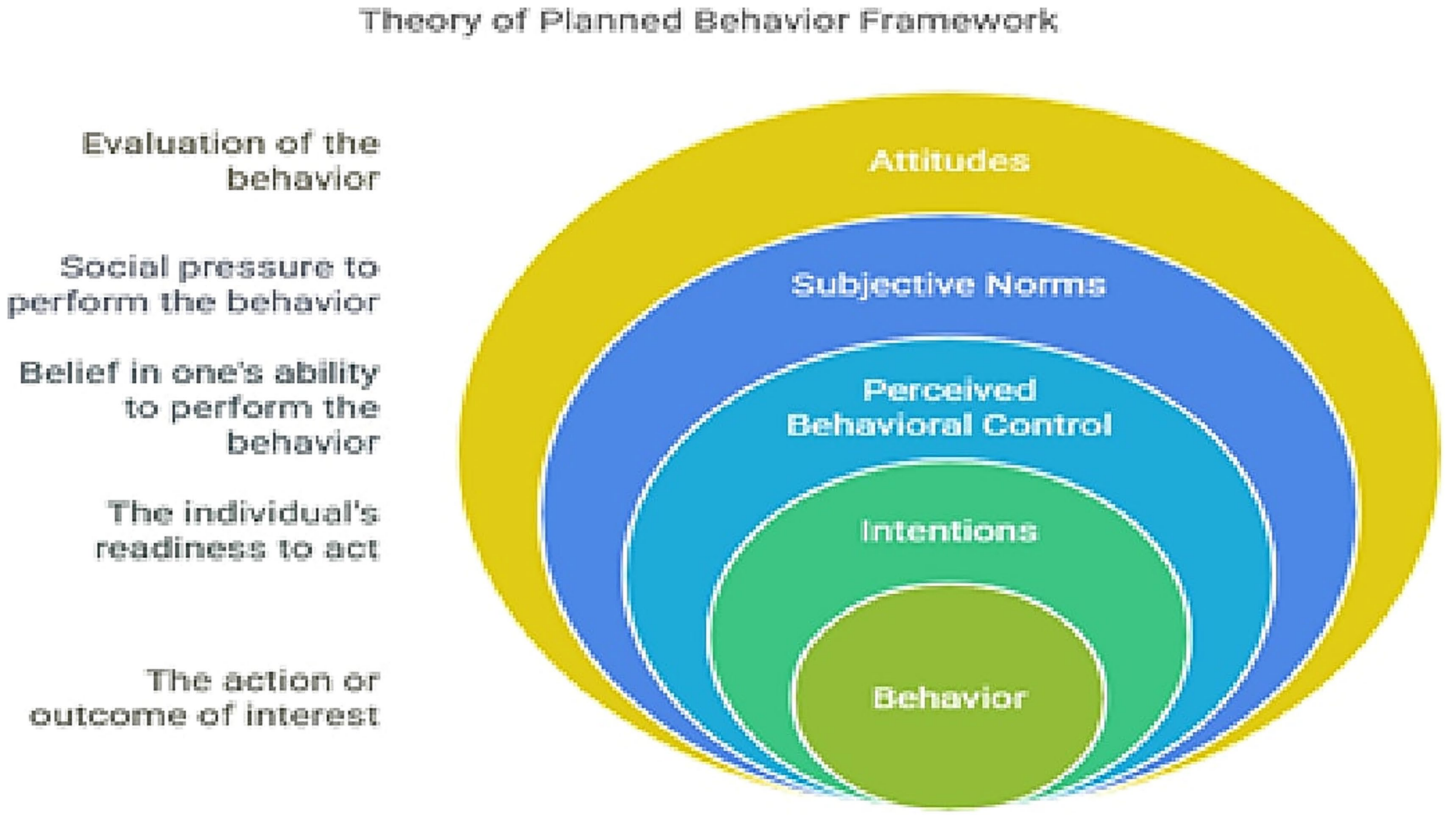
Analyzing food safety and obesity challenges.

Figure 7 depicts the interconnected issues of obesity and food safety, highlighting how reactionary governance and regulatory shortcomings, such as those evident during the BSE crisis, expose consumers to both short-term foodborne dangers and long-term metabolic hazards. In addition to providing a visual chronology of significant public health incidents, the figure also functions as a conceptual map that connects structural food system reform with crisis-driven regulation. The necessity of shifting from incident-triggered policy reactions to anticipatory and systems-level governance that considers marketing strategies, consumer behavior, and nutrition research is emphasized. As our understanding of the need for integrated regulatory supervision to address food safety and public health has grown, so has the inclusion of microbiological and chronic disease risks in the same framework.

The changing media landscape, particularly social media, necessitates a more sophisticated comprehension of its dual function in influencing eating habits. Although traditional journalism can be a valuable tool for public health, unregulated nutrition messages have become widely disseminated through platforms like YouTube, Instagram, and TikTok. Research shows that 95–98% of diet and health-related posts on social platforms are either false or lack scientific accuracy (67). Influencers and content producers frequently use platform algorithms to increase reach by promoting energy-dense, nutrient-poor (EDNP) foods under the pretense of health and wellness. In addition to reinforcing commercial food narratives, this environment makes distinguishing between reliable facts and marketing content difficult, creating new difficulties for regulatory supervision and consumer protection. As a result, policy frameworks need to go beyond traditional media to cover digital spaces and platform-based advertising, especially to protect young people and those with poor literacy levels from widespread false information (68, 69). Fat or food industry propaganda becomes part of a daily diet of information, successfully diverting attention away from the highly profitable energy-dense, nutrient-poor products, ironically leaving consumers ignorant of the proper nutrition needed to maintain and cope with obesity costs and public initiatives (70).

### 4.1 Current regulatory frameworks in food advertising

The issue regarding childhood nutrition and health has focused much of its interest on the promotional activities of the food (and soft drinks) industries. Global organizations like the World Health Organization have asked national governments to review the controls they exercise over the marketing efforts of these industries (71). The food sector has a significant and controversial influence on dietary guidelines and public health initiatives. By lobbying, supporting research that supports their interests, and opposing regulations that jeopardize profit margins, large food and beverage companies have been shown to significantly impact governmental policies. Evidence-based remedies like sugar levies, front-of-pack labeling, or limitations on marketing to children are frequently diluted, delayed, or blocked due to this corporate political effort. At the same time, governmental entities have occasionally failed to implement strict consumer protections, whether due to inadequate regulatory capacity, political considerations, or economic constraints (44). Therefore, the intricate relationship between state institutions and private sector interests is crucial in maintaining the obesogenic environment, calling for greater accountability, transparency, and policy processes that put public health first.

Various promotion methods in food advertising enhance awareness, liking, and intention to eat foods, and almost all the food marketing promotions are unhealthy. Food ads emphasize taste, enjoyment, and satisfaction, making self-control challenging and provoking hunger or food thoughts. Appealing food packaging and marketing at sales points can induce impulse buying (72). The United Kingdom’s (UK) government office overseeing communications, the Office of Communications, introduced new rules in January 2007 that limit the kinds of food advertised to children. They are established through nutrient profiling by the Food Standards Agency (FSA) and exclude all foods high in fat, sugar, and salt from promotion to children (73–75). China is the world’s largest food producer and consumer. From the early 1980s, the agro-food industry has experienced phenomenal growth across the entire food supply chain, from agricultural production to trade, agro-food processing to food retailing, and food service to advertising and promotion (76).

A thorough summary of the legal frameworks controlling food advertising in various jurisdictions and their effects on consumer behavior is given in this paragraph. Under the General Food Law, Regulation (EC) No 178/2002 in the European Union establishes stringent food safety and marketing guidelines, emphasizing traceability, transparency, and avoiding false claims, particularly about health benefits. It acts as the cornerstone of regulations in all EU member states to guarantee consumer protection. In the United States, the FDA (Food and Drug Administration) and FTC (Federal Trade Commission) jointly regulate food advertising. The FDA regulates food labeling and nutrition claims, and the FTC keeps an eye on advertising tactics to stop misleading marketing, particularly regarding children and other vulnerable populations. This two-pronged approach seeks to guarantee that food marketing’s health-related messaging is truthful and not deceptive.

Independent food regulation was formed in the UK after Brexit by organizations including the Food Standards Agency (FSA), Department for Environment, Food and Rural Affairs (DEFRA), and Ofcom. The UK has implemented stringent laws to prevent the promotion of unhealthy foods, especially high-fat, salt, and sugar (HFSS) products, to children through media outlets (77). There is some regulatory ambiguity, particularly in cross-border advertising, due to the post-Brexit transition. Together, these regulatory frameworks represent a variety of intersecting approaches to regulating food marketing and safeguarding public Health. Companies frequently modify their marketing to comply with or circumvent these restrictions, even though they serve as guidelines for how food is marketed and seen. Understanding these frameworks is essential to developing international regulations that uphold moral business conduct and safeguard consumers.

### 4.2 Proposed policy interventions

National governments and global food industries are leading the food environment and diets producers. Governments are responsible for ensuring healthy food environments and promoting healthy choices to enhance health, promote the environment, and minimize inequalities (78). The function of underlying institutions and the impact of synergies from other programs and policies affect how effective a program or policy intervention is. The function of underlying institutions and the effect of synergies from different programs and policies both affect how effective a program or policy intervention is (79). Legume production in Europe is limited by barriers due to system lock-ins and capacity deficits in the food system. Systematic analyses refer to low production and consumption because of several causes: (a) farmers’ ignorance about the non-marketed value of legumes, (b) agri-environmental policies and payments not internalizing the negative externalities of crop specialization, (c) lower and uncertain yields resulting in lower profitability compared to non-legume crops, (d) insufficient access to independent agricultural extension services for legume systems, (e) missing capacities for aggregation and post-treatment, and (f) problems of classification with legumes among wholesalers (80–82). Evidence indicates that encouraging fruits, vegetables, wholegrains, nuts, and fish and limiting animal fats, trans fats, and salt could save millions of lives. Reducing salt and substituting trans fats with polyunsaturated fats are the WHO’s “best buys” in preventing non-communicable diseases, which they have described as cost-effective, cheap, achievable, and acceptable (78).

**Figure 8:**
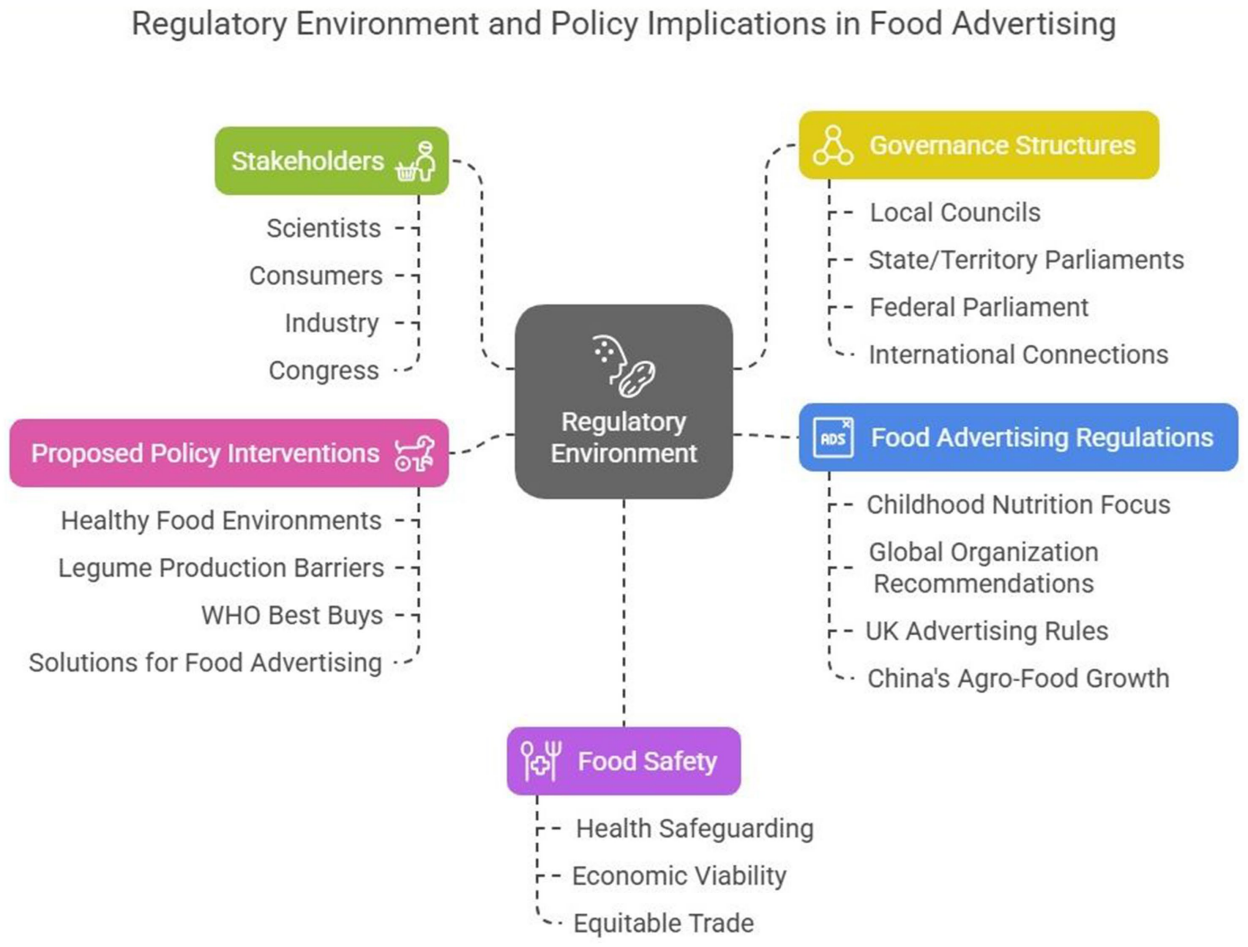
Regulatory environment and policy implications in food advertising.

Policymakers and the scientific community have proposed a wide range of solutions that could potentially mitigate the adverse effects of using cartoon characters in food advertising (83). These include general labeling legislation, targeted advergames bans, further calls for radically reforming nutrient profiles, changing corporate behavior and/or using legal regulatory tools such as banning certain food advertising during children’s programming slots, and focusing concern on the promotion of foods that contribute to childhood overweight and obesity or unhealthy eating habits (83) (Figure 8).

### 4.3 Policy delays in England and the role of nutrient profiling

England has continually postponed the introduction of full limitations on TV (Television) and internet marketing for foods high in fat, sugar, and salt (HFSS), while taking the lead in the early efforts to limit food advertising to children. The proposed 9 p.m. watershed restriction and curbs on paid online advertising were implemented in 2022 as part of the 2020 “Tackling Obesity” policy. Still, they have subsequently been repeatedly delayed and are now anticipated to take effect in 2025. The effectiveness of public health policy is called into question by this ongoing delay, which also reflects the industry’s growing influence on regulatory decisions. The urgency of combating childhood obesity is undermined by these delays, which allow susceptible audiences to continue being exposed to deceptive advertising for nutrient-poor items. This kind of exposure makes health disparities worse, particularly for low-income groups that are disproportionately impacted by circumstances that promote obesity (84).

The Ofcom Nutrient Profiling Model (NPM), created by the Food Standards Agency (FSA) to categorize foods suitable for advertising to children, is a crucial tool in the UK’s policy toolbox. By weighing “negative” components (such as saturated fat, carbohydrates, and sodium) against “positive” components (including fiber, protein, fruit, and vegetable content), the model assigns a score to products depending on their nutritional makeup. The scientific soundness and practicality of this evidence-based paradigm have earned it recognition on a global scale. Its delayed implementation, however, indicates a conflict between political desire and scientific consensus.

Significantly, these delays raise concerns about regulatory capture, the process by which influential food and advertising organizations shape state regulations, and they point to a larger issue with the implementation of nutrition programs: the conflict between public health goals and economic motivations. NPM-based advertising limitations must be enforced promptly and without compromise to achieve the desired health goals, given the proven association between HFSS advertising and childhood obesity, particularly through priming effects and behavioral signals (85).

## 5 Ethical considerations in food marketing

Ethics is the rightness or wrongness of action. Organizations are ethical when they act ““the right thing” and unethical when they act “the wrong thing (86).” In Today’s food production, transportation, processing, wholesale, and retail chain, food may be mismanaged or exposed to chemicals or microbiological diseases, making food safety a moral dilemma. Consumers worldwide fear food because of the well-known food scandals of the last several decades, such as the BSE, melamine, dioxin, avian flu, or H1N1 occurrences, and concerns about various residues, chemicals, antibiotics, and hormones in food. There are several ethical concerns with the production of raw materials, the raising of animals, and the manufacturing and marketing of food. However, we have not addressed the ethical issues in the food and agriculture sciences comprehensively and institutionally (86–88). Ethical considerations of food marketing activities are essential, as children are particularly susceptible to marketing influences. Advertising messages may negatively influence children’s food choices, purchase requests, and food consumption and encourage actions to decrease that influence (89, 90). Despite this position, the food and beverage industry continue to use food advertisements prominently featuring such enticements as free giveaways, promotional product tie-ins, and third-party licensed characters, sponsorships of popular children’s programs, and product placements in movies and television programs watched by children and adolescents (91).

### 5.1 Deceptive advertising practices

Beyond the sheer volume of food marketing, the nature of promotional messages has raised significant concerns about the food industry’s motives. Advertisers commonly use well-established persuasive strategies that disproportionately impact vulnerable groups, particularly youngsters, such as emotional appeals, inflated health claims, and cartoon-based endorsements. These strategies have become even more pernicious as marketing moves toward digital and interactive formats, making it difficult to distinguishing between promotion and entertainment. The UK horse meat controversy (2013) illustrates the wider societal repercussions of false advertising. When it was discovered that several beef items from well-known British and European supermarkets contained unreported horse meat, the public was incensed, and concerns over traceability, label accuracy, and regulatory enforcement were raised. The controversy exemplifies how deceptive product representation, whether in packaging, claims, or ingredient disclosures, can undermine customer trust and call for strict control procedures, even though it is not strictly a typical advertising failure. It emphasized systemic flaws in supply chain verification. It illustrated how important proper marketing is to safeguarding public health (92). Similarly, functional food advertising in Asia, such as for spreads that lower cholesterol or immune-boosting drinks, frequently uses pseudo-scientific terminology to selectively promote benefits while leaving out dose information or legal warnings. 85% of health-related ads made false promises, according to a thorough survey conducted by the Hong Kong Consumer Council. These techniques take advantage of regulatory weaknesses and consumer health concerns, leading to the “health halo effect,” which exaggerates the benefits of a product while misrepresenting its overall quality. Other examples include “no sugar added” drinks that contain a lot of concentrated fructose or “low-fat” snacks packed with artificial additives and salt. Although these statements are lawful under loose labeling regulations, they deflect consumers’ focus from the overall quality of their diets and encourage uninformed consumption habits (93).

In addition to functional foods, misleading marketing techniques are standard in goods carrying health claims such as “immune-boosting” drinks (94) and “cholesterol-lowering” spreads, which, under EU regulation (EC) No 1924/2006, are classified as health claims and require prior scientific authorization. These claims frequently conflate food and medication by exploiting regulatory gaps and consumer health concerns. Despite efforts by regulatory frameworks like the EU’s Regulation (EC) No 1924/2006 on nutrition and health claims to require scientific verification, marketers usually use vague language or selectively provide facts to deceive customers about the effectiveness of a product. A margarine product, for example, might contain plant sterols that have been shown to decrease cholesterol. Still, the true benefit varies greatly depending on dosage and dietary context information, which is not often revealed in advertising. The same applies to “low-fat” or “no sugar added” items, which may seem healthy but include significant harmful substances, such as artificial additives or processed carbs. Customers may overestimate the health benefits of a product and overindulge in it as a result of these activities, which can produce a “health halo effect.” In the end, this kind of advertising damages consumer confidence and jeopardizes public health initiatives by diverting focus from the quality of whole diets to single-product fixes.

**Figure 9:**
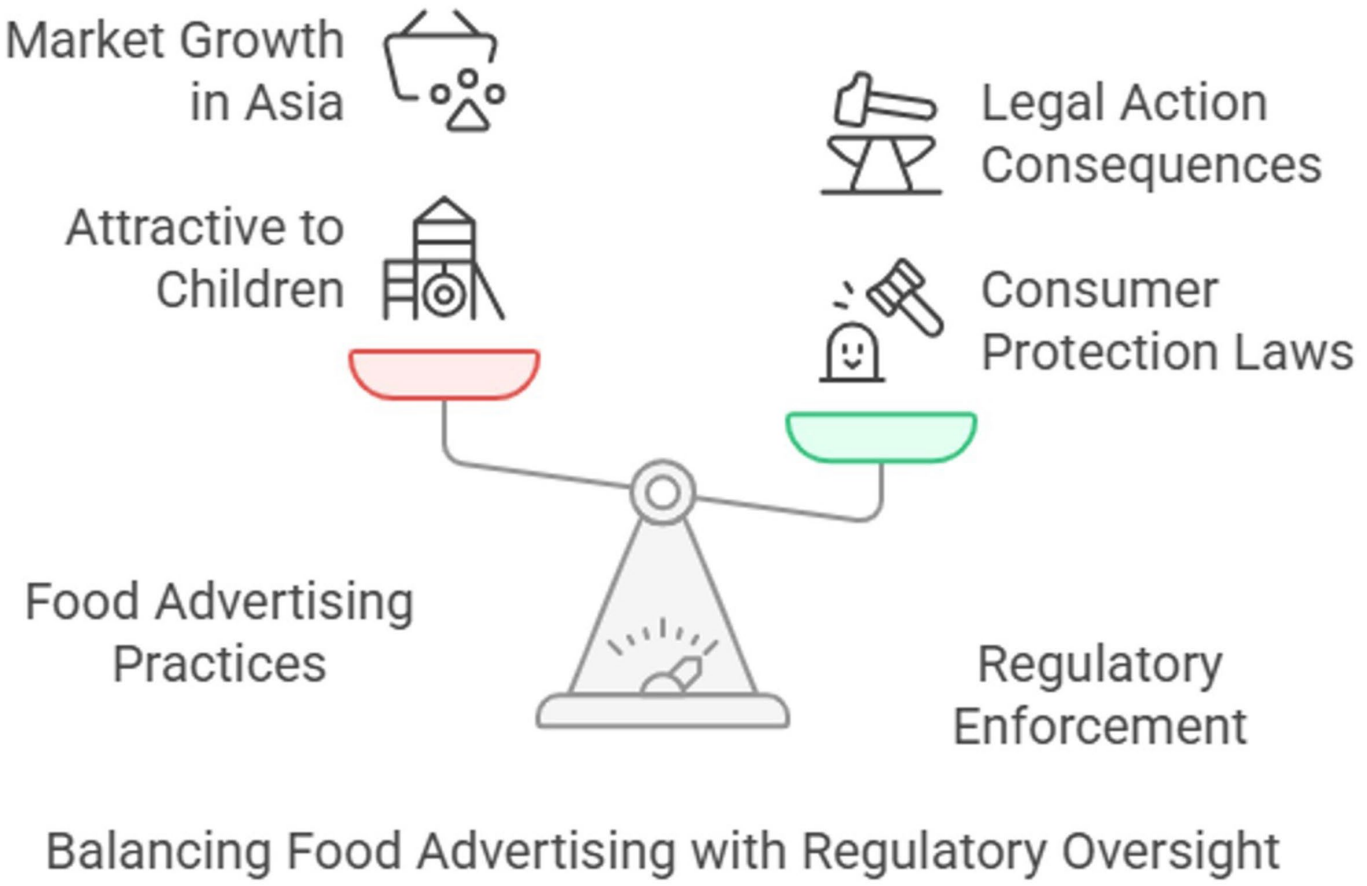
Balancing food advertising with regulatory oversight.

Regulatory agencies like the European Food Safety Authority (EFSA) and the Federal Trade Commission (FTC) are responsible for stopping such misleading advertising. However, enforcement is still uneven, particularly in different jurisdictions with different standards. Class-action lawsuits, shareholder litigation, and civil penalties have all been used to hold businesses responsible, but proactive, unified international policies are still desperately needed (95–97) (Figure 9).

### 5.2 Promoting healthy choices

One of the key aims of health promotion is to simplify the process of people making healthier choices. One of the key concepts here is empowerment. Health professionals have a remit to facilitate and enable individuals toward empowerment (98). Food selection is underpinned by conscious thought and automatic, habitual, and unconscious processes (99). Point-of-choice cues are volitional or post-intentional aids to health behavior since they take effect following a decision by people to better their health; the cue reminds people of a previous intention and ensures that the window of opportunity is not lost. In isolation, a cue will not affect behavior; it must precede an intention to change (99, 100). Food-based dietary guidelines (FBDG) are consistent and easily understandable translations of population nutrient goals to encourage healthy habitual food choices and improve public health. Establishing and implementing national/regional FBDG has the potential to provide considerable health and economic dividends. FBDG were initially designed to counteract nutrient-deficiency disease, but they could potentially have a significant role in deterring/promoting the uptake of specific dietary habits, which have been linked to preventing chronic non-communicable diseases (CNCD; e.g., CVD, some cancers) (101, 102).

**Figure 10:**
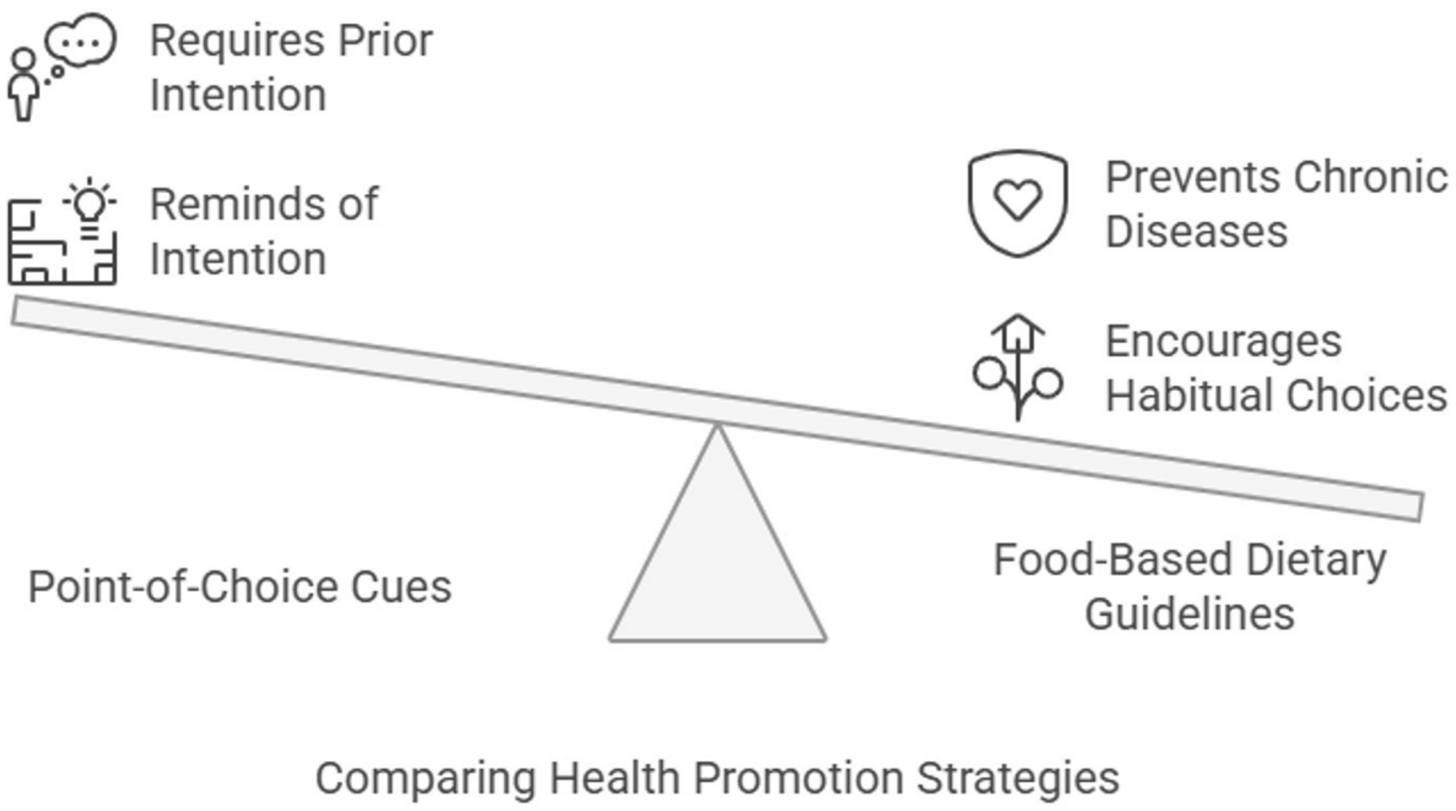
Comparing health promotion strategies.

What is considered a healthy diet is still being determined by science. There is no longer a recommended daily limit for cholesterol in the 2015–2020 and 2020–2025 Dietary Guidelines for Americans. Citing a lack of data to establish a dose–response link between dietary cholesterol and blood cholesterol levels, they instead recommend consuming as little dietary cholesterol as possible while maintaining a nutritionally adequate diet (103) (Figure 10).

## 6 Research methodologies in studying consumer choices

The consumer may be thought of as an imperfect problem solver. Consumer behavior toward food products is purposeful, but the consumer is constrained by limitations of information, cognitive ability, memory, and time (104). Research and consumer education on the dangers of food safety malpractices are crucial to reducing foodborne illness since a significant portion of food preparation and handling occurs in the home (105, 106). Finding out how consumers handle food in their homes, what they know about food safety, and why some safe food-handling procedures are followed while others are not have been the goals of consumer food safety research. Providing information for creating successful communication techniques to encourage safe food-handling behaviors has been the overarching goal of most studies (106–108). Global diets have also been drastically altered by the rise of ultra-processed foods (UPFs), which are industrially manufactured goods high in added sugars, fats, salt, and additives. Child-targeted ads, convenience messages, and emotional appeals extensively promote these products. UPFs are commonly available but nutritionally inadequate since they are frequently cheap, shelf-stable, and heavily promoted. Higher UPF intake has been connected in numerous studies to an increased risk of cardiovascular disease, metabolic syndrome, and obesity. Because they are essential conduits for the formation and reinforcement of unhealthy eating habits, discussions of food marketing and obesity must take into consideration both the widespread marketing of UPFs and portion size manipulation.

Studies have shown that observed food-handling practices of trained food handlers can be superior to those of consumers, and, hence, findings of studies related to qualified individuals from the food trade were not included because they might skew general outcomes and conclusions in the review. Other studies excluded included those primarily focused on risk perception of different areas of food safety, like pesticide residues or bovine spongiform encephalopathy (BSE) (106). There is a lengthy history of consumer food preferences and quality perceptions. However, in recent years, these topics have received increased attention as a result of the intense debate over such issues as ethical considerations concerning food production and quality, food scandals and the resulting consumer food scares, genetic modification of foods, and animal welfare (or, rather, non-welfare), which has made questions about food quality and consumers’ supposedly rational or irrational food choices even more pressing. Increased interest in health and quality stands in striking contrast to a perceived unwillingness to pay the higher prices implied, and suspicion about industrial food production stands in opposition to busy lifestyles and a resultant need for convenience (109, 110). A thorough search of the prior literature was conducted to uncover published and unpublished consumer food safety studies. Electronic searches of computerized library databases and screening of reference lists from pertinent research papers and reports made it easier to identify numerous published studies. Internet browsers were used to search the World Wide Web, and several unpublished foreign studies were obtained through the “Foodsafe” list serv. Attending international food safety conferences and personal conversations with specialists in the area resulted in collecting the results of numerous unpublished research studies (106). The bovine spongiform encephalopathy (BSE) crisis of the 1990s was one of the most significant turning points in the development of European food regulation. Essential flaws in the current regulatory framework, such as dispersed authority, inadequate risk communication, and inadequate traceability throughout the food supply chain, were made clear by this food safety disaster. As cases of variant Creutzfeldt-Jakob Disease (vCJD), a human neurological disease associated with BSE, started to appear, public confidence in food governance at the national and EU levels quickly declined (111).

**Figure 11:**
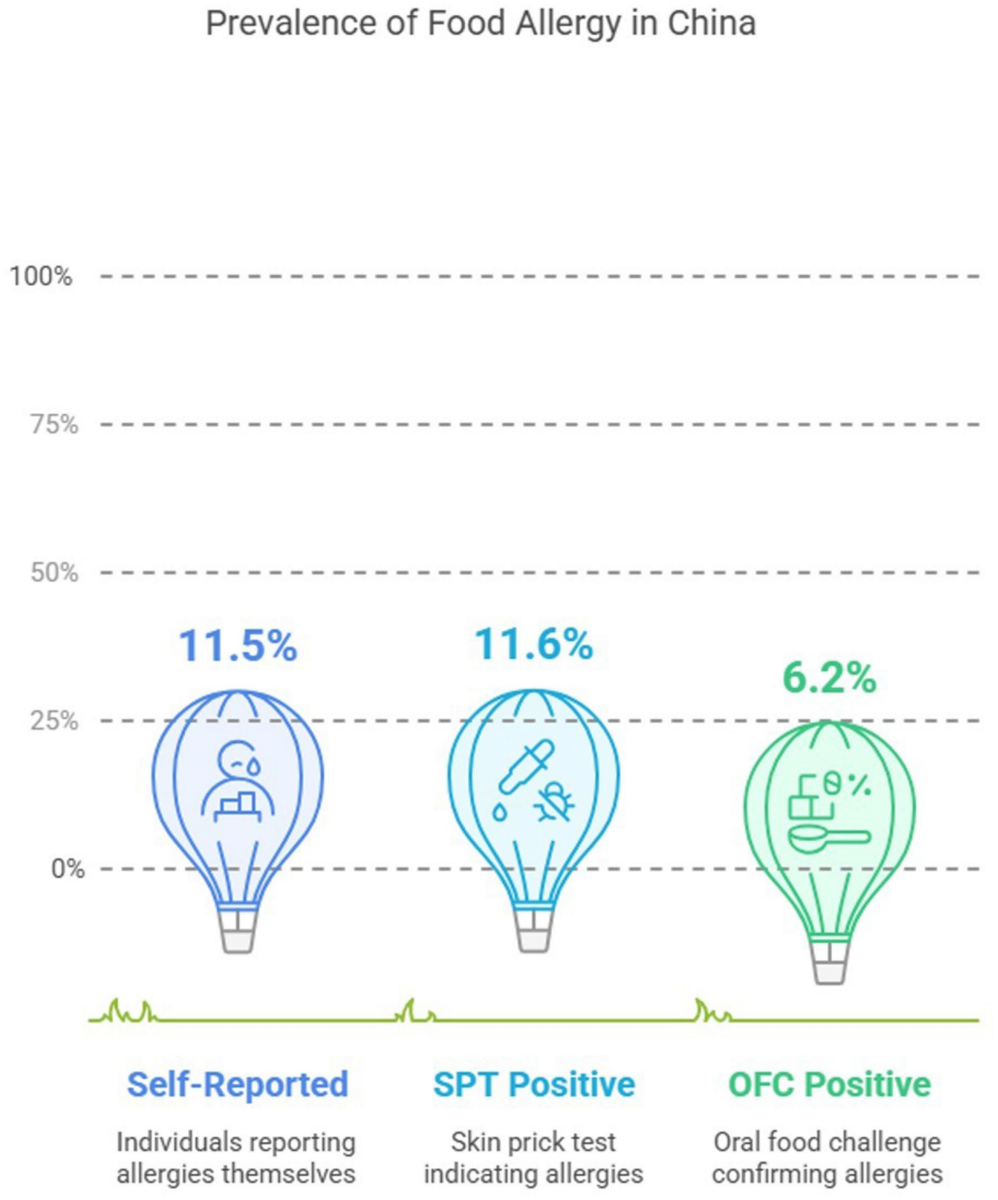
Literature search process for food safety studies.

The European Union responded by completely revamping its framework for food safety. Adoption of Regulation (EC) No 178/2002, also referred to as the General Food Law, was the most significant result. This rule created the European Food Safety Authority (EFSA) and established the precautionary principle, food traceability, and risk communication transparency as the cornerstones of all later food laws. By ensuring that food marketed in the EU is safe, ethically, and scientifically validated, these policies represent a paradigm change from reactive to preventive food regulation. The BSE crisis was a regulatory turning point that influenced the current structure of EU food regulation, making it more than just a historical oddity. It is a critical case study of how public health crises can lead to systemic changes in governance and policy-making, changing how risks are identified, shared, and handled in food marketing (112) (Figure 11).

A cross-disciplinary referencing approach is used in this narrative review to guarantee a thorough comprehension of the intricate connection between food marketing and obesity. We purposefully incorporated empirical research from consumer marketing, behavioral science, and nutrition, well-known theoretical frameworks (such as the Theory of Planned Behavior and Social Cognitive Theory), and international policy literature. This integrative approach improves the review’s scholarly depth and practical relevance while promoting a well-balanced synthesis of views.

Analyzing how governments and regulatory agencies have responded to safeguard consumers—especially children—from deceptive food advertising is essential after examining aggressive marketing tactics. Policymakers have implemented various regulatory measures, from statutory interventions to voluntary norms. While certain areas have seen improvement, regulatory enforcement is still uneven, underscoring the need for more thorough and empirically supported policy frameworks worldwide.

### 6.1 Experimental studies and field research

Most traditional direct experimental studies and field research have either examined the impact of TV advertising on children’s food preferences, consumption patterns, or choice behavior toward junk foods’ or have examined only specific food groupings relevant to food standards regulations (36, 113, 114). The advertised foods mirror the eating pattern linked to childhood obesity and dental caries risk and are contrary to health authority guidelines [e.g., (115)] that children and young people should be encouraged to eat an extensive range of nutrient-dense foods (e.g., fruits, vegetables, cereals, lean meats, dairy foods) and to restrict intake of foods high in fat and sugar, the term used here to describe junkor unhealthy food (113, 115). The impact of digital microtargeting, affective priming, and visual signals on food choices in natural environments is being examined in increasing experimental research. According to research employing eye tracking, fMRI, and online behavioral monitoring, digital platforms can customize food marketing to appeal to subconscious reward systems rather than logical analysis. These impacts are particularly noticeable on social media sites like YouTube, TikTok, and Instagram, where exposure to food-related content is linked to higher snack frequency and lower dietary quality. Additionally, cross-sectional and longitudinal studies show that personal characteristics, including impulsivity, health literacy, and length of media exposure, impact emotional and cognitive reactions to food advertisements. These results lend credence to a move toward behavioral models informed by neuroscientific research that consider consumer choice’s emotive and autonomic aspects in obesogenic environments. Regarding nutritional knowledge, food advertising has little impact on children’s overall perceptions of a healthy diet. However, in some contexts, it does affect more specific forms of nutritional knowledge. For instance, exposure to soft drink and cereal adverts lowered primary-aged children’s capacity to decide correctly whether or not a given product included real fruit (116, 117).

## 7 Case studies and real-world impacts

In the Latin American and Caribbean (LAC) region, rampant child and adult obesity, unhealthy and insufficient physical activity are driving high rates of diabetes, high blood pressure, and other non-communicable diseases (NCDs) (118–120). Conversely, massive proportions of the population in many countries in the region are undernourished and stunted from poor nutrition early in the 1^st^ 1,000 days of their lives. Contemporary food systems influence LAC demand and supply via midstream and downstream processing and wholesale, retail, and transportation practices (119, 120). These are integrated with liberalization, privatization, foreign investment, infrastructure investment, and urbanization. Modernized procurement systems and the coevolution of these sectors supply large processors, supermarkets, and fast-food chains. Consequently, urban and rural LAC regions undergo rapid and pervasive change (120).

**Figure 12:**
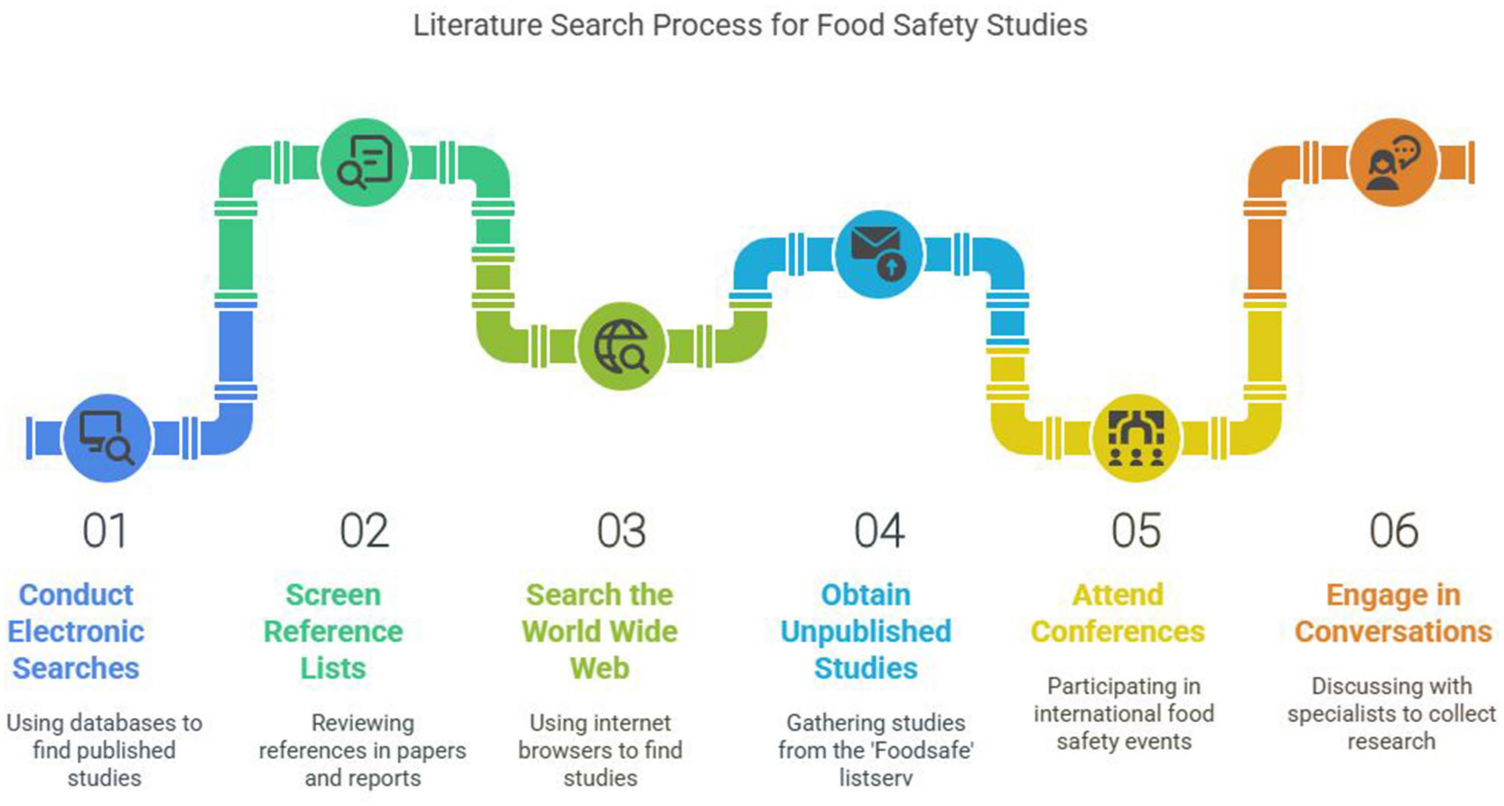
Prevalence of food allergy in China.

New York City’s ban on trans fats was followed by changes in consumer purchasing patterns toward higher-calorie non-trans-fat-free replacements. In response to calls to eliminate sugar-sweetened beverages in stores, a pilot program was announced to offer price incentives for purchasing fruits and vegetables in various combinations. Participants in the pilot said they did not notice a change in their purchases, consistent with instances in which healthy food promotion has not enhanced consumer sales in supermarkets (121, 122). No nationwide epidemiological survey has been conducted in China, resulting in a paucity of understanding regarding key allergens among the Chinese allergic population. However, a meta-analysis found that the average prevalence of food allergy for self-reported was 11.5, 11.6% for SPT positive, and 6.2% for oral food challenge positive (OFC-positive) (123, 124) (Figure 12).

### 7.1 Fast food industry practices

The fast-food sector provides consumers easily and time-conveniently available food products due to effective production technologies. Traditionally, fast food places are chains of franchised establishments selling identical food products like hamburgers, pizzas, chicken, or sandwiches on the menu (125). As childhood obesity and overweight rates rise across the world, more research is needed on the economic, political, and social factors that impact children’s diets. The rise of fast food in LMICs, such as China, India, Brazil, Nigeria, and Pakistan, shows ‘ow aggressive marketing changes the food environment to encourage reliance on highly processed, branded meals. Because of their aspirational branding and low prices, fast-food restaurants are usually found close to schools and in low-income metropolitan neighborhoods. Transnational firms can use sophisticated digital campaigns, special pricing, and celebrity endorsements without significant public health remedies due to the absence of strong advertising rules. As a result, dietary shifts in LMICs are characterized by the normalizing of unhealthy consumption patterns through effective marketing techniques and the increased availability of unhealthy items. The dual burden of malnutrition—the cohabitation of stunting and obesity—is greatly influenced by this marketing-driven change, especially for young people in metropolitan areas (126). In 2016, the World Health Organization (WHO) reported that 39% of adults had obesity, whereas the obesity rate in 1975 was only around 3% in men and 6% in women (127, 128).

Fast-food restaurants (FFRs) are an environmental element, since they primarily provide meal facilities (other than snack and non-alcoholic drink bars), where customers often order or choose products and pay in advance to eat. FFRs facilitate fast food consumption, which frequently contains significant calories, saturated fat, trans fat, sugar, simple carbohydrates, and salt, at a cheap cost (129). Food marketing targeted toward children before the 1970s consisted mainly of breakfast cereals and packaged meats like hot dogs and cold cuts, as well as TV advertisements. Fast food was still an emerging fast-growth industry with shops all across America. Its brand presence in the form of locations was but a fraction of its current representation (130).

### 7.2 Success stories in healthier food marketing

Recent European case studies (131) have revealed adequate commercial food and beverage marketing initiatives encouraging a wider range of demographic groups to adopt healthier eating habits. Their success, however, was not only attributable to their branding strategy or product kind, as a careful analysis shows. Instead, three crucial enabling factors, public trust, regulatory alignment, and integration with larger nutrition literacy initiatives, were necessary for effectiveness. The Nutri-Score front-of-pack labeling system, for instance, was widely adopted by consumers in France and Belgium, primarily due to institutional support, open scientific methodology, and consistent media messaging. Comparable traffic-light programs, on the other hand, failed in different countries because of a lack of consumer awareness or opposition from those involved in the food sector. These instances demonstrate that “success” in marketing healthy foods necessitates multifaceted cooperation between the media, civic society, and policymakers in addition to visually appealing rebranding. Furthermore, cultural sensitivity is essential: tactics that appealed to Nordic customers by highlighting environmental sustainability might not translate well to contexts where taste or price are the primary motivators for purchases. Replicating these results necessitates considering local socioeconomic and psychological conditions and campaign technical design. Dual-system decision models, which take into account both automatic (such as the visual prominence of labels) and conscious (such as nutrition literacy) processes in food choices, are very important, according to researchers studying “food well-being.” Even though they may initially interest consumers, advertising that cannot bridge various cognitive systems frequently has no lasting effect. Future interventions must therefore establish reinforcing settings that modify default choices through persistent availability, pricing, and visible mechanisms, in addition to communicating healthier options (132–134).

The General Food Law (Regulation (EC) No 178/2002) establishes the European Union’s consumer protection and food safety framework. It strongly embraced traceability, transparency, and the “precautionary principle,” which states that food marketing cannot deceive customers, particularly when making claims about nutrition or health. With severe consequences for infractions, all communications about food must be precise, factual, and evidence-supported. Member states also use these guidelines when advertising to minors and other vulnerable populations. Food labeling and marketing are heavily regulated by the Food and Drug Administration (FDA) in the United States. The FDA enforces laws like the Nutrition Labeling and Education Act (NLEA), which mandates that food goods show American nutritional information. The Federal Trade Commission (FTC) also looks for dishonest advertising tactics, particularly in kid-friendly television (135).

During lockdowns, many nations temporarily relaxed marketing prohibitions and nutrition labeling requirements, which resulted in a spike in online delivery promotions and digital food advertising. Understanding these regulatory frameworks is crucial to comprehending the larger sociopolitical context of food policy since they substantially impact the dynamics of food marketing and its effects on public health.

## 8 Future trends and innovations in food marketing

Innovations in digital personalization, nanotechnology, and biotechnology will progressively influence the next generation of food marketing. From a public health standpoint, these technologies present both opportunities and risks. For example, nanotechnology has made nutrient encapsulation, smart packaging, and improved food safety via nanosensors possible (136). By extending shelf life and optimizing nutrient delivery, these advances can increase the accessibility of healthier food. But in the absence of strict regulatory control, these technologies could be appropriated to improve the shelf life and palatability of highly processed foods, which would encourage obesogenic eating habits (137). Nutraceuticals and functional foods are frequently positioned as remedies for lifestyle or age-related illnesses. Still, they are also increasingly advertised with health claims that could conflate medicine and nutrition. In jurisdictions where regulatory restrictions on substantiating such claims are inadequate, this presents ethical and policy issues. According to the evidence, the quality of a whole diet may suffer if customers rely too much on these “health-enhanced” goods. This leads to a disjointed view of nutrition that prioritizes specific items over dietary trends (138).

Another significant area of innovation is digital advertising. Food marketers can now hyper-personalize product placements on social media, gaming platforms, and even wearable fitness applications thanks to machine learning and big data analytics (139), While it is theoretically possible to utilize precision targeting to encourage healthy behaviors, present commercial practices mostly target young people and socioeconomically disadvantaged groups with energy-dense, nutrient-poor items. Because individualized digital exposure is frequently linked to impulsive intake and a decreased ability to regulate one’s diet, health disparities worsen. Furthermore, the rate at which these technologies are developing is significantly faster than the rate at which related governance and ethical frameworks are being developed. Because of this, public health organizations are dealing with an increasing regulatory lag, in which policies cannot keep up with the rapidly changing commercial food settings. Transdisciplinary cooperation between behavioral science, ethics, law, and digital technology will be necessary to close this gap (140).

In conclusion, how technologies are regulated, interpreted, and incorporated into larger public health objectives will ultimately determine the direction of food marketing and the technology. The same instruments that have the potential to transform health outcomes may instead strengthen commercial drivers of diet-related disease if a conscious effort is not made to tie innovation to nutritional justice and transparency (140, 141).

**Figure 13:**
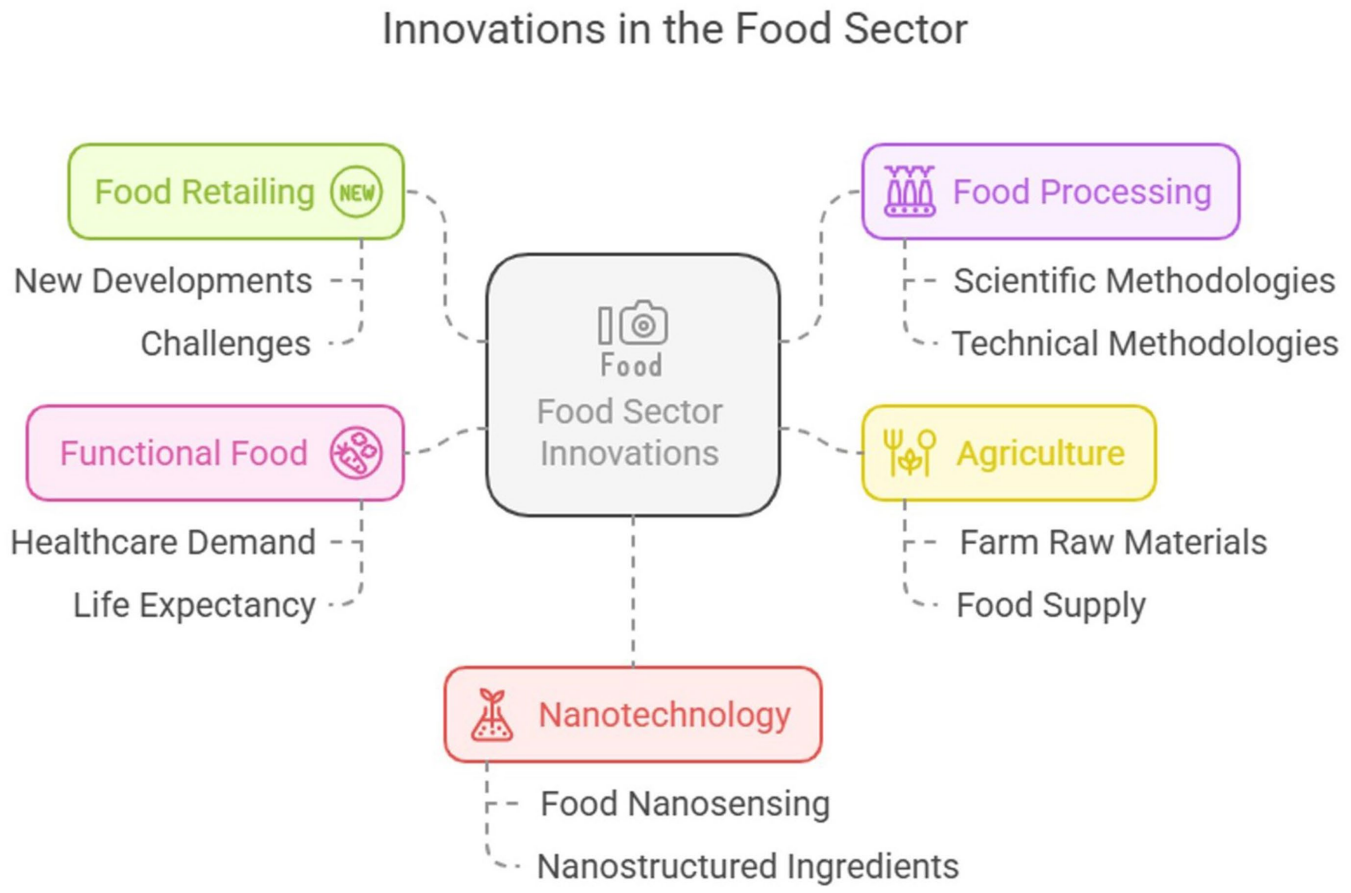
Food sector innovations.

Emerging technical advancements in the food industry are shown in Figure 13, such as innovative packaging, AI-based automation, and nanotechnology. Although integrating these innovations into marketing ecosystems presents new challenges, they also present exciting avenues for enhancing food safety, nutrient delivery, and sustainability. Dietary self-regulation is made more difficult by the use of nanoscale encapsulation, which can improve nutrient bioavailability while also intensifying the sensory attraction of EDNP meals. In a similar vein, marketing powered by AI can hyper-target customers with deceptive ads while also personalizing dietary recommendations. This image thus captures a fundamental paradox: depending on the ethical stance, openness, and regulatory vision of the institutions involved, technological innovation in the food industry can either reduce or worsen obesogenic settings. This dual approach encourages critical thinking on the trade-offs between public health and the digital transformation of food marketing.

## 9 Emerging technologies in advertising

Historically, the food industry has never been technology-driven. But over the last decade, drastic changes have been made due to the shifting demographics and changing lifestyle of the consumer; the public health policies that were influencing these lifestyle changes; the expanding fitness food market, a byproduct of these changes; and the food industry’s new aggressive stance toward adoption of cutting-edge technologies to meet consumer demands (142). Using social media for marketing has enabled companies to share more product information, target specific audiences, customize messages, and redefine advertising to include communication, education, and entertainment, blurring the distinction between commercial and non-commercial content (143).

Research on food advertising focuses on food television (TV) and demonstrates a significant frequency of unhealthy food commercials (ads). In Brazil, over 90% of all food adverts announced on open TV are from ultra-processed foods, and 96% used one or more persuasive advertising tactics (144). A significant percentage of T’Today’s 12- to 17-year-olds play games online. Online gaming has become the most popular feature on family-oriented platforms. Researchers are now saying that because older school-aged children spend so much time online, food companies can influence what they eat by strategically advertising online games. Socially oriented games often depict activities surrounding food and drink, and provide tremendous advertising opportunities for food marketers. Advertisers are turning to virtual advertisements for sports-related gameplay to reach children who may not be playing kid-oriented games. One beverage brand was the first to do this with its product placed in a sports-oriented game. So far, researchers and public health advocates are concerned that there are no rules or guidelines to which companies advertising virtual products must adhere, and virtual advertising does not require parental consent to reach children (51, 145, 146).

## 10 Conclusion and recommendations

We have explored the impact of food marketing on obesity. We have endeavored to cast a wide net and take a more general look at marketing practices. Consumer concerns and economic considerations dictate that food quality, safety, and health all be considered along with traditional food pricing issues and consumer price expectations. The economic theory, without question, indicates that the current relatively free availability of high-calorie, less expensive, and exceedingly tasty food is likely one of the significant driving forces that have led to the high prevalence of obesity. It is clear that food marketers are not, nor can they be expected to, implement public policies. It is also clear that when developing and implementing public policy, we must recognize the economic underpinnings of those decisions. In the past, we have maintained and offered here that a certain level of freedom at the individual consumer level, coupled with the vibrant forces of our free economy, works to our strength and success.

If we are to navigate in the right direction, we are called upon to inform and protect consumers from themselves. In addition, we must structure both market and non-market choices accordingly. Solving the rise in the obesity rate will not be found in the easy focus on one aspect, nor will it be solved with a single public policy solution that tries to sell such a focus. Any program of attempts will require a more multifaceted range of considerations than a single solution will allow. Consequently, we provided an economic overview of the collective factors at play, which led to a list of considerations we have displayed.

Combining knowledge from public Health, nutrition science, marketing theory, psychology, and behavioral economics, this review crosses disciplinary boundaries to thoroughly examine the intricate relationship between food marketing strategies and obesity. We provide a comprehensive understanding of how food advertising, branding, and policy environments influence individual dietary choices and, ultimately, population-level health outcomes by projecting theories of consumer behavior (such as the Social Cognitive Theory and the Theory of Planned Behavior) onto modern marketing strategies and their physiological and social repercussions. This multidisciplinary synthesis offers a strategic framework for creating more successful, health-promoting interventions and enables a detailed understanding of the systemic causes of obesogenic environments.

## 11 Recommendations for future research directions

To extend the previous discussion, future researchers in food marketing and obesity at both the macro and micro levels could conduct resampling studies exploring the complex underpinnings and reciprocity of the relative influences that genetics, individual characteristics, social environment, and technological changes have on obesity trends. Furthermore, it would be beneficial for professionals and researchers to have validated tools to identify the growing disparities that are emerging with specific regard to whom to target, when to intervene, the manner of intervention, the length, and the level of intervention. Next, other longitudinal studies could address areas of food marketing beyond the ones focused on in this chapter. One particular area could be the influence of food marketing at different points in people’s socio-economic paths. It needs to be made relevant by understanding what point in the life cycle and what the underlying mechanisms could be. Specifically, researchers could target children at different stages in their life and their nutrition and physical activity behaviors. This, in particular, would be useful to the academic and policy-making communities.

Following health behavior change, the goal would be to provide insights that might generate information that professionals and researchers could use to design unique and innovative strategies for promoting health to a segment of the population that is currently difficult to reach –low-income families. At present, obese people trying to lose weight must contend with a variety of negative influences. Without a supportive environment, attempts at behavioral change are destined to be difficult and likely to fail. If society is committed to reducing the prevalence of obesity, perhaps more should be dedicated to modifying social values and norms, which have helped create the problem. There is no panacea for reversing the complexity of the obesity trend; however, policymakers should consider strategies in all settings—social, physical, and economic—and begin increasing public awareness that obesity can be curbed, and help spread this message.

## 12 Limitations of the narrative review approach

In order to properly contextualize the findings and suggestions, it is necessary to note a number of methodological constraints, even though this review offers a thorough and multidisciplinary synthesis spanning public health, behavioral science, marketing theory, and nutrition policy. While narrative reviews are a good way to organize cross-disciplinary and conceptually complex content, they do not have the rigorous quality appraisal and repeatability standards that systematic reviews have. This raises the possibility of selection bias because conceptual relevance, not predetermined risk-of-bias criteria, was used to choose which papers should be included. Despite using a variety of databases and gray literature sources, the generalizability of some findings may be impacted by variations in study design, population, and quality. Moreover, the lack of a systematic methodological evaluation restricts our capacity to consistently evaluate the strength of the evidence that is included. Although the synthesis’s interpretive value is not diminished by these constraints, they do highlight the necessity of cautious extrapolation, especially when it comes to policy implications and future research possibilities. While acknowledging the need for further empirical, longitudinal, and intervention-based research to bolster the body of evidence and confirm the conceptual frameworks developed here, readers are urged to interpret the recommendations within the parameters of a narrative methodology.
